# sp^2^/sp^3^–Hybridized nitrogen–mediated electrochemical CO_2_ capture and utilization

**DOI:** 10.1126/sciadv.adw6592

**Published:** 2025-06-20

**Authors:** Zhenfang Zhang, Yitong Li, Yiwen Zhong, Peng Li, Lingfeng Zhu, Zhi Zheng, Baohua Jia, Matthew David, Yang Fu, Hai Yu, Tianyi Ma

**Affiliations:** ^1^ Centre for Atomaterials and Nanomanufacturing (CAN), School of Science, RMIT University, Melbourne, VIC 3000, Australia.; ^2^ School of Textile Science and Engineering, Xi’an Polytechnic University, Xi’an 710048, China.; ^3^ ARC Industrial Transformation Research Hub for Intelligent Energy Efficiency in Future Protected Cropping (E2Crop), Melbourne, Australia.; ^4^ CSIRO Energy, 10 Murray Dwyer Circuit, Mayfield West, NSW 2304, Australia.; ^5^ GrapheneX Pty Ltd., Level 3A, Suite 2, 1 Bligh Street, Sydney, NSW 2000, Australia.

## Abstract

Electrochemical carbon dioxide (CO_2_) capture and utilization, powered by renewable energy, are essential to achieving net-zero emissions and CO_2_ valorization. While remarkable progress has been made in catalysts, solution design, and system engineering, recent breakthroughs reveal that nitrogen-containing molecules—specifically sp^2^-hybridized structures (e.g., pyridine) and sp^3^-hybridized moieties (e.g., ethanolamine) —hold untapped potential to revolutionize both CO_2_ capture and conversion. These structures have been demonstrated as the Holy Grail in facilitating CO_2_ activation, stabilizing key intermediates, and streamlining reaction pathways—capabilities rarely achievable with conventional strategies. However, limited mechanistic understanding of their physicochemical properties and interactions with CO_2_ hampers broader application. This review highlights recent advances in leveraging sp^2^/sp^3^-hybridized nitrogen structures, unpacks their molecular roles in electrochemical CO_2_ management, and offers a unifying framework for their dual-functionality across capture and conversion. By illuminating these nitrogen-based motifs, we uncover practical design principles and open avenues for integrating expanded N-containing compounds into energy technologies—paving the way for next-generation carbon management strategies.

## Teaser

sp^2^/sp^3^–Hybridized nitrogen structures revolutionalize electrochemical CO_2_ capture and conversion.

## INTRODUCTION

The heavy reliance on fossil fuel resources (e.g., coal, natural gas, and crude oil) has caused carbon dioxide (CO_2_) emissions of up to ~1500 gigatonnes in the past decades and led to soaring atmospheric CO_2_ concentration over 420 parts per million (ppm) in 2024 (*1*). High-emitting industries including ammonia production, cement manufacturing, iron and steel making, and petroleum refining contribute ~40% of global emissions (*2*). These emissions have exacerbated climate challenges, such as rising global temperatures and extreme weather events, underscoring the urgent need for effective solutions. Carbon capture and/or utilization technologies have emerged as promising approaches to mitigate postcombustion emissions while transforming CO_2_ into valuable products (*3*). When integrated with renewable energy sources like solar and wind power, these technologies offer a “plug-and-play” opportunity to decarbonize energy-intensive industries while simultaneously generating industrial feedstocks. Among the available methodologies, electrochemical approaches stand out as state-of-the-art solutions because of their high energy efficiency, precise control over reaction rates, ambient operating conditions, compatibility with renewable energy, and cost-effectiveness. These techniques are also highly versatile, seamlessly integrating with other technologies and accommodating applications ranging from pilot-scale studies to full-scale industrial implementation. Electrochemical carbon capture and conversion can be broadly categorized into two pathways, electrochemical CO_2_ capture and electrochemical CO_2_ conversion, distinguished by their carbon management strategies. To be specific, typical electrochemical CO_2_ capture pathways include the following: (i) metal-amine coupling (*4*), (ii) redox-active molecular interactions (*5*), (iii) pH swing via proton-coupled electron transfer (*6*) or membrane-mediated processes (*7*), and (iv) capacitive adsorption (*8*). Considerable efforts have been focused on various aspects, ranging from mechanism investigation to system engineering, including process efficiency optimization (*9*), tuning of molecular redox potentials (*10*), screening of capture agents (*11*), and comprehensive reviews of the electrode, electrolyte, and membrane (*12*). On the other hand, electrochemical CO_2_ conversion primarily focuses on traditional gas-phase CO_2_ electrolysis and integrated CO_2_ capture and conversion processes (*13*). Research in this field has explored innovative catalyst designs, manipulation of local reaction environments, direct reduction of dilute CO_2_ streams, formation of high-value multicarbon products, and advanced system engineering (*14*, *15*).

**Fig. 1.**
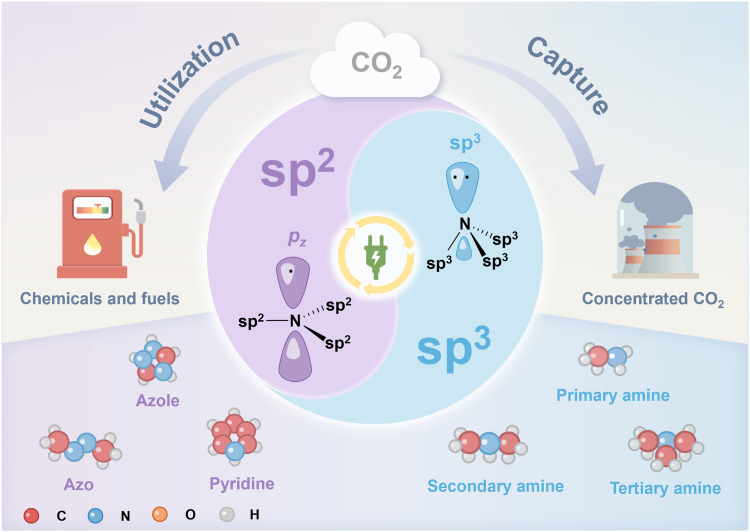
sp^2^/sp^3^–Hybridized nitrogen structures in the mediation of electrochemical carbon capture and conversion.

Notably, a common thread across these systems is the involvement of sp^2^/sp^3^–hybridized nitrogen structures, which play a pivotal role in electrochemical CO_2_ capture and conversion processes (Fig. 1). This observation inspired us to delve deeper into the unique properties of these hybridized nitrogen centers and their functions. To this end, from the viewpoint of hybridized nitrogen centers, we provide a concise overview of sp^2^/sp^3^–hybridized nitrogen for electrochemical CO_2_ capture and conversion, which is outlined as follows. First, we discuss the fundamental properties of sp^2^-N and sp^3^-N compounds including their definitions, classifications, and interactions with CO_2_. Then, we summarize the sp^2^-hybridized nitrogen–mediated electrochemical CO_2_ capture methodology, covering the reaction mechanism, strategies for enhancing capture efficiency, and system optimization. Subsequently, we highlight sp^3^-hybridized nitrogen–mediated electrochemical CO_2_ capture and reactive CO_2_ capture, emphasizing the rationale behind absorbent design, authentic reactant species, electrode development, electrolyte modification, and electrolyzer engineering. In the following, we investigate sp^2^/sp^3^–hybridized nitrogen–mediated gas-phase CO_2_ reduction, focusing on its contributions to adsorption and mass transfer, proton delivery regulation, and cocatalytic effects. Last, we identify key scientific challenges and propose future research directions to accelerate progress in this field. Through this review, we aim to provide a holistic understanding of how sp^2^/sp^3^–hybridized nitrogen structures can advance electrochemical CO_2_ capture and conversion technologies, ultimately paving the way toward industrial-scale applications.

## SP^2^/SP^3^–HYBRIDIZED NITROGEN STRUCTURES

### Definitions and classifications

In this context, sp^2^/sp^3^–hybridized nitrogen (sp^2^/sp^3^-N) specifically implies a series of molecules or compounds with nitrogen-containing functional groups bearing at least one sp^2^ or sp^3^ hybridization structure. Ammonia (NH_3_) and its derivatives (e.g., amines) are typical representatives of sp^3^-hybridized nitrogen structures, while pyridine, phenazine (PhN), and imidazole are a class of sp^2^-N compounds. The sp^3^-N structure endows amines with an additional lone pair of electrons, which could be donated to other electron-deficient molecules (e.g., CO_2_) via nucleophilic substitution reactions. Amines are generally categorized as primary, secondary, and tertiary amines depending on the number of hydrogen atoms attached to the nitrogen atom (*16*). Also, amines can be classified into mono- or multiple amines and linear chain or cyclic chain amines according to the number of amino groups and the carbon chain structures on the amines (*17*). sp^2^-N structures have much lower basicity or nucleophilicity compared to sp^3^-N amines because sp^2^-N hybridization has a higher proportion of s orbital character that holds electrons closer to the nucleus. Consequently, the lone pair of electrons remains tightly bound to the nucleus, making it unlikely to participate in bond formation. In aromatic systems, the lone pair of electrons also contribute to the formation of aromatic sextet that the electrons are very stable and hardly involved in a bond formation, which would also disrupt the system’s stability. On this basis, the sp^2^-N compounds are usually involved in the bond formation in the premise of additional electron transfer.

### sp^2^/sp^3^-N─CO_2_ interactions

**Fig. 2.**
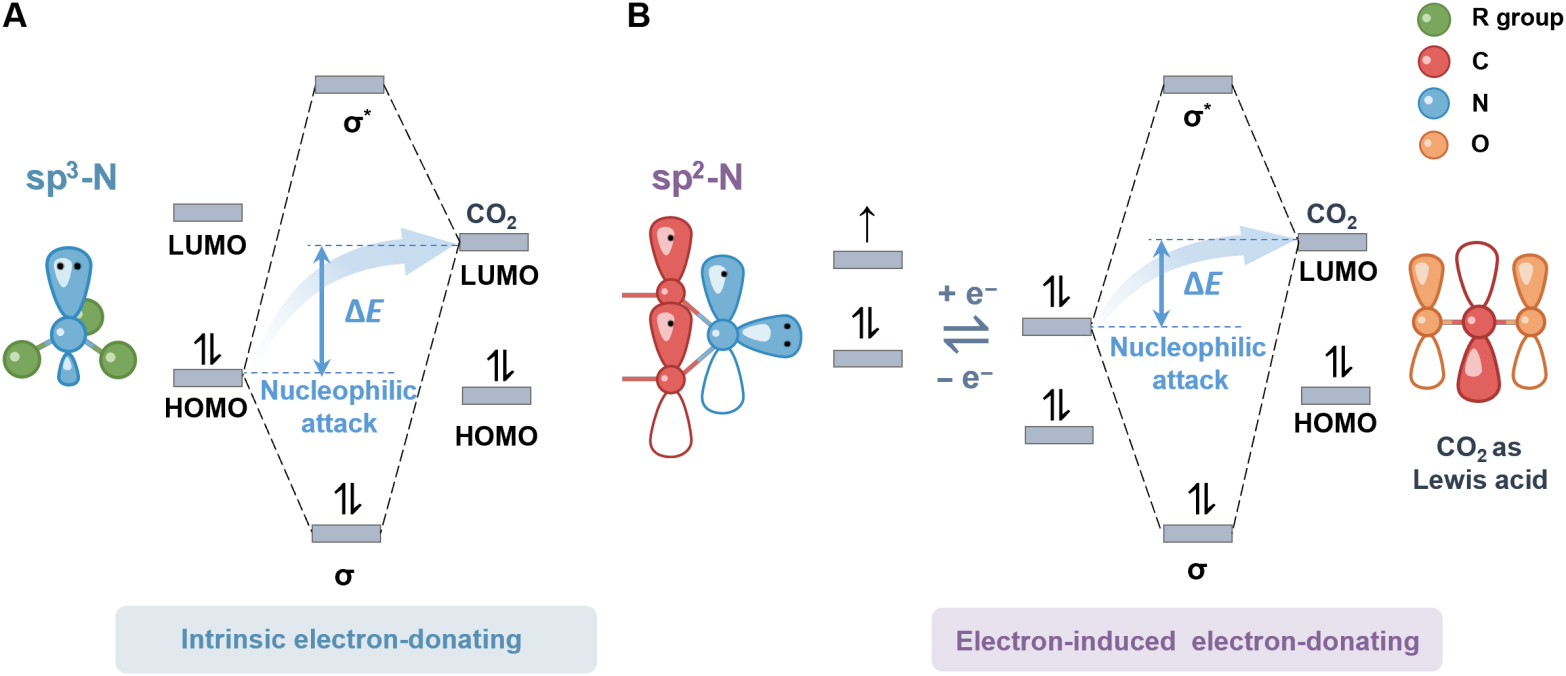
Electronic interactions of sp2/sp3-N structures with CO_2_. (**A**) sp3-N and (**B**) sp2-N.

This section presents how the sp^2^/sp^3^–hybridized nitrogen structures interact with CO_2_ molecules in the presence/absence of an additional electron-inducing process. sp^3^-N structures like amines have lone pairs of electrons that can readily attack electron-deficient CO_2_ molecules, as this process occurs spontaneously without external current/voltage intervention. For primary/secondary amines (Eqs. 1 to 3), chemical absorption first dominates because of its rapid reaction rate. As equilibrium is approached and free amines are depleted, carbamate hydrolysis leads to bicarbonate and carbonate formation, with physical absorption gradually prevailing, resulting in a much slower kinetics (*18*). Overall, the reaction mechanism involves zwitterion formation, proton transfer, deprotonation, and carbamate hydrolysis (Fig. 2A) (*19*). This explains why aqueous amine solutions often exceed their theoretical CO_2_ loading capacity based on the stoichiometric amino group–to–CO_2_ ratio. For tertiary amines, they act as bases and catalyze CO_2_ hydration to form protonated amine and bicarbonate instead of carbamate. Thus, tertiary amines can be considered intermediates for converting free CO_2_ to bicarbonates (*16*). Hindered amines, such as 2-amino-2-methyl-1-propanol (AMP), are a distinct class of amines with bulky functional groups that disrupt the formation of stable carbamates, distinguishing them from conventional primary and secondary amines. The steric hindrance created by these substituents near the nitrogen weakens the N─H and N─C bonds, facilitating carbamate hydrolysis and promoting the formation of protonated amines and (bi)carbonate, similar to the behavior of tertiary amines (*19*). Therefore, the capture capacity of hindered amine solvents is usually higher than that of primary and secondary amines.

$$\text{Primary amines}:{\text{2RNH}}_{2}+{\text{CO}}_{2}\to {\text{RNH}}_{2}{\text{COO}}^{–}+{{\text{RH}}_{3}}^{+}$$ (1)

$$\text{Secondary amines}:{\text{2R}}_{1}R_{2}\text{NH}+{\text{CO}}_{2}\to R_{1}R_{2}{\text{NHCOO}}^{–}+{{\text{RH}}_{3}}^{+}$$ (2)

$$\text{Tertiary amines}:R_{1}R_{2}R_{3}N+{\text{CO}}_{2}+H_{2}O\to R_{1}R_{2}R_{3}{\text{NH}}^{+}+{{\text{HCO}}_{3}}^{–}$$ (3)

For sp^2^-hybridized nitrogen, the lone pair on N is delocalized because of conjugation with the π-system of the C═N bond. This delocalization reduces the electron-donating ability of N by lowering the energy of its lone pair. A highest occupied molecular orbital and lowest unoccupied molecular orbital (HOMO-LUMO) analysis shows that the HOMO of the sp^2^-N center is at a lower energy level compared to the LUMO of CO_2_, making direct adsorption or reaction between sp^2^-N compounds and CO_2_ unfavorable under most conditions (Fig. 2B) (*20*, *21*). Thus, through electrochemical regulation, introducing electrons into the p-orbital of sp^2^-N can generate another lone pair with a higher energy level, enabling effective CO_2_ sorption. On the basis of this characteristic, sp^2^-N compounds can selectively adsorb and release CO_2_ under electrochemical conditions (Eq. 4)

$${\text{sp}}^{2}-N:R_{1}═N─R_{2}+{\text{CO}}_{2}+e^{–}\to R_{1}─N─\left({\text{COO}}^{–}\right)─R_{2}$$ (4)

Detailed cases of sp^2^/sp^3^-N structures, classifications, and their CO_2_ interaction properties are shown in Table 1.

**Table 1.** Fundamental properties of sp^2^/sp^3^–hybridized nitrogen structures and their roles in electrochemical CO_2_ capture and conversion.

sp^2^/sp^3^ structures	Classifications	Name	Chemical structure	CO_2_ loading (mol CO_2_/mol sp^2^/sp^3^-N)	Roles in carbon capture/conversion	Ref.
sp^3^-N (amine)	Primary amine	Monoethanolamine (MEA)	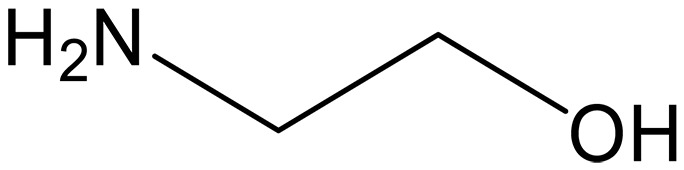	0.51–0.54 (30 wt %)	Capture agents	(*93*, *102*, *103*, *137*, *146*)
					Capture agents for ICO_2_CE^*^	
					Inhibitor/promoter for HER	
		2- Amino- 2- methyl- 1- propanol (AMP)	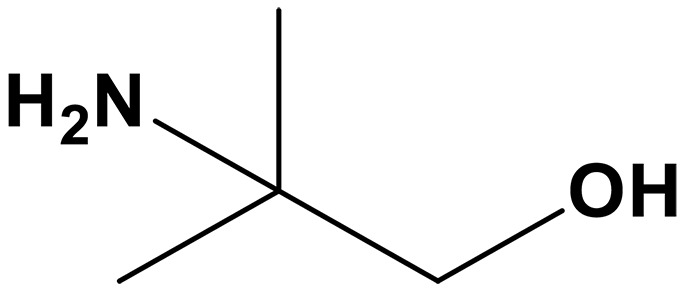	0.59–0.8 (30 wt %)	Capture agents for ICO_2_CE^*^	(*88*, *89*)
		2-ethoxyethyl- amine (EEA)	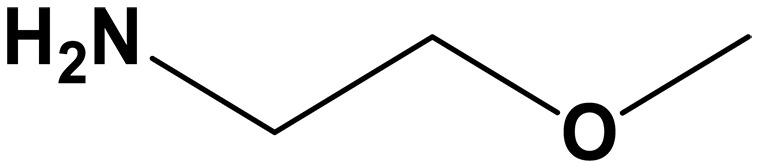	0.4–0.6 (30 wt %)	Capture agents for ICO_2_CE^*^	(*92*)
		Ethylenediamine (EDA)	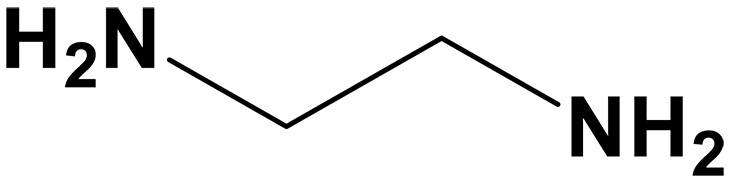	0.48 (12 M)	Capture agents for ICO_2_CE^*^	(*60*)
					Capture agents for EMAR^†^	
		4-aminobutylphos phonic acid (NH_2_BPA)	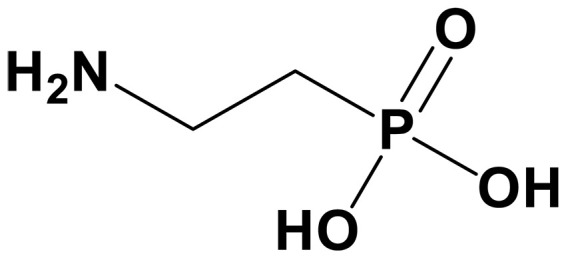	0.3–0.5 (30 wt %)	Molecular ligand to enhance CO_2_ adsorption	(*140*)
	Secondary amine	Diethanolamine (DEA)	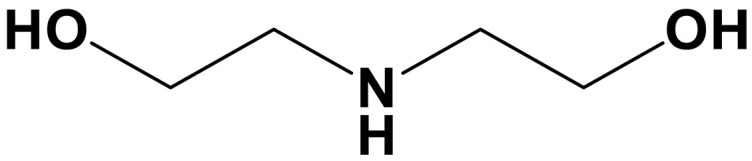	0.6–0.65 (3 M)	Capture agents for ICO_2_CE^*^	(*13*, *127*)
					Molecular ligand to enhance CO_2_ adsorption	
		Piperazine (PZ)	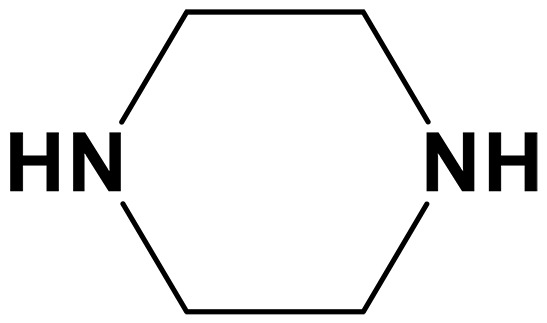	0.26 (40 wt %)	Cocatalytic effect	(*128*)
	Tertiary amine	Methyldiethanolamine (MDEA)	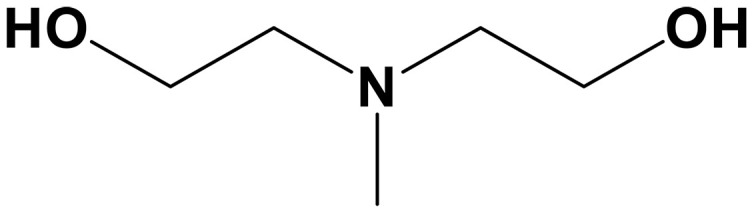	0.39 (30 wt %)	Capture agents for ICO_2_CE^*^	(*78*)
		Amine-linked COFs	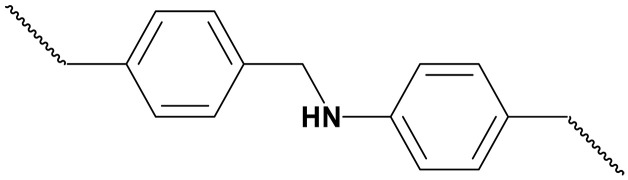	–	Amine-linked COF to enhance CO_2_ adsorption	(*147*)
		Trimethylamine (TREA)	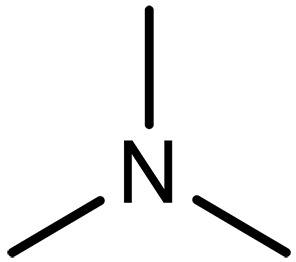	0.89 (2 M)	Capture agents for ICO_2_CE^*^	(*90*)
		1-Cyclohexylpiperidine (CHP)	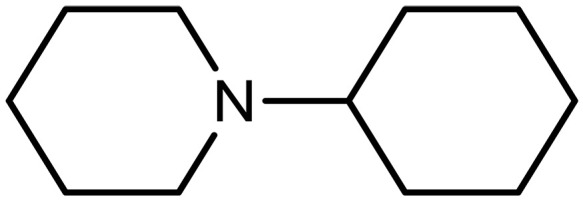	0.3–0.5 (30 wt %)	Capture agents for ICO_2_CE^*^	(*87*)
	Polyamine	Polyamine	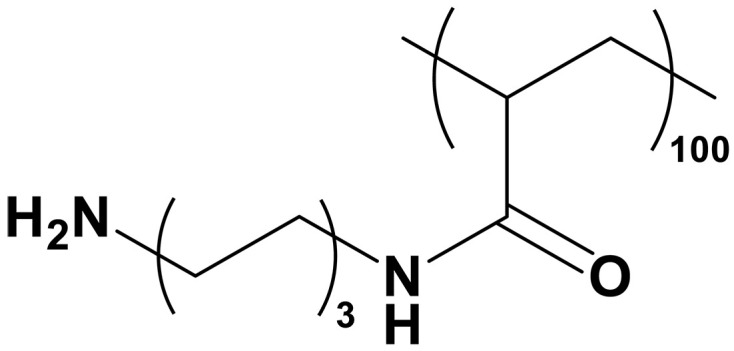	–	Cocatalytic effect to activate intermediate	(*130*, *142*)
sp^2^-N	Azole	Imidazolium	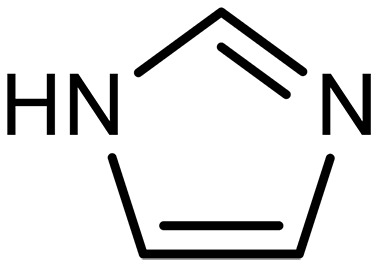	–	Cocatalytic effect to facilitate CO_2_ transport	(*131*)
		Poly[(3-methyl-1-vinylimidazoliummethylsulfate)-*co*-(1-vinylpyrrolidone)] (PQ44)	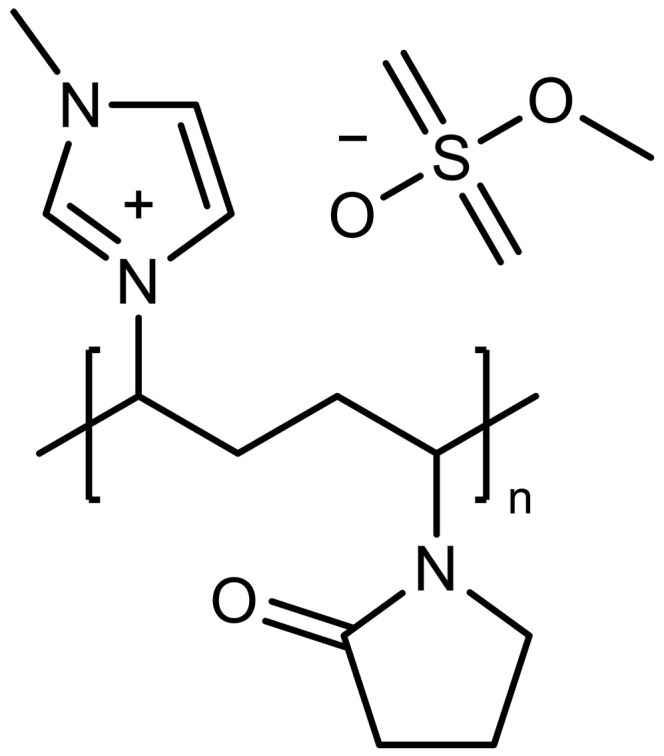	–	Cocatalytic effect to facilitate CO_2_ transport	(*132*)
		2-amino-5-mercapto-1,3,4-thiadiazole (AMT)	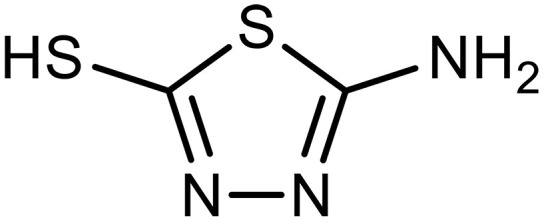	0.3–0.5 (30 wt %)	Molecular ligand to enrich local CO_2_ concentration	(*148*)
	Pydrine	4,4′-Bipyridine	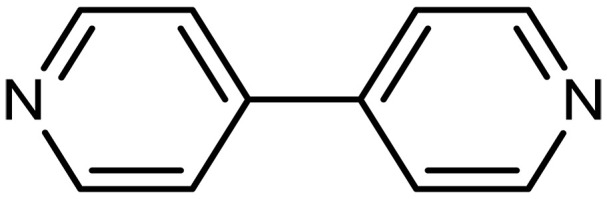	1^§^	Capture agents for EMCC^‡^	(*148*)
		1-Aminopyridinium nitrate	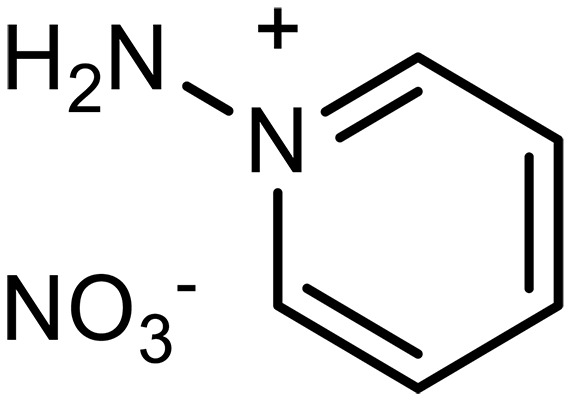	2^§^	Capture agents for EMCC^‡^	(*35*, *148*)
	Azo	Azopyridine	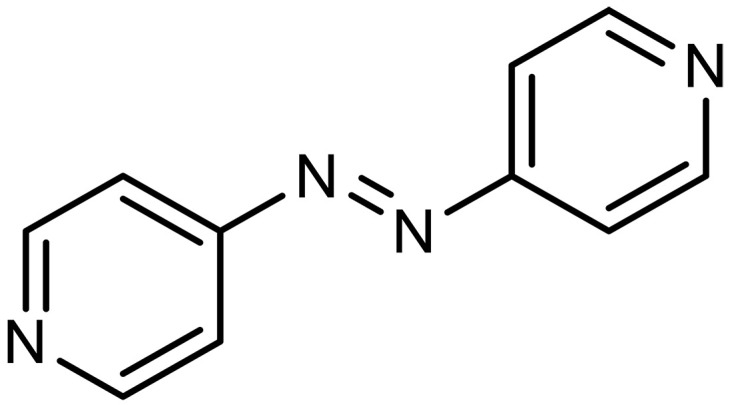	2^§^	Capture agents for EMCC^‡^	(*20*, *148*)
	Phenazine	Phenazine	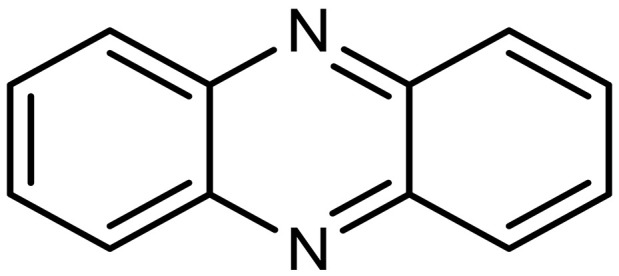	2^§^	Capture agents for pH-swing EMCC^‡^	(*25*, *45*, *148*)

*ICO_2_CE means integrated CO_2_ capture and release.

†EMAR means electrochemically mediated amine regeneration.

‡EMCC means electrochemically mediated carbon capture.

§The CO_2_ capacity of the capture agent in the EMCC process differs from that of the spontaneous reaction in amines; only the theoretical maximum value is provided here.

## SP^2^-N–MEDIATED ELECTROCHEMICAL CO_2_ CAPTURE

In this section, we present the advanced electrochemical CO_2_ capture technology mediated by sp^2^-N compounds such as pyridine, PhN, etc. The concept of electrochemical CO_2_ capture originated in the 1980s with the use of molten carbonates as electrolytes (*22*). Subsequently, it evolved into electrochemically mediated carbon capture (EMCC), relying on redox-active species to coordinate with CO_2_ as the core mechanism. As discussed above, sp^2^-N demonstrates limited binding affinity for CO_2_ because of its reduced nucleophilicity. However, under a reduction current, its interaction with CO_2_ is enhanced, enabling efficient CO_2_ capture. Conversely, under oxidative conditions, the nucleophilicity of sp^2^-N diminishes, promoting the release and enrichment of CO_2_. This process, known as sp^2^-N–mediated electrochemical CO_2_ capture, involves the adsorption and enrichment of gaseous CO_2_ through the electrochemical modulation of sp^2^-N structures, which function as direct or indirect capture agents. Powered by renewable electricity, electrochemical CO_2_ capture offers a sustainable solution to concentrate CO_2_ from a range of dilute sources, including flue gases and air. Compared to conventional thermal swing technology, it reduces capital expenditure, streamlines the operation process, and improves energy efficiency, making it a highly promising carbon capture technology (*20*, *23*). Building on this foundation, we tentatively categorize this sp^2^-N–mediated electrochemical CO_2_ capture into direct and indirect (pH-swing) configurations. The distinction lies in the binding interactions of sp^2^-N species: Direct capture involves binding directly to CO_2_, while indirect capture depends on binding to H^+^ (*4*, *24*).

### sp^2^-N–mediated direct CO_2_ capture

**Fig. 3.**
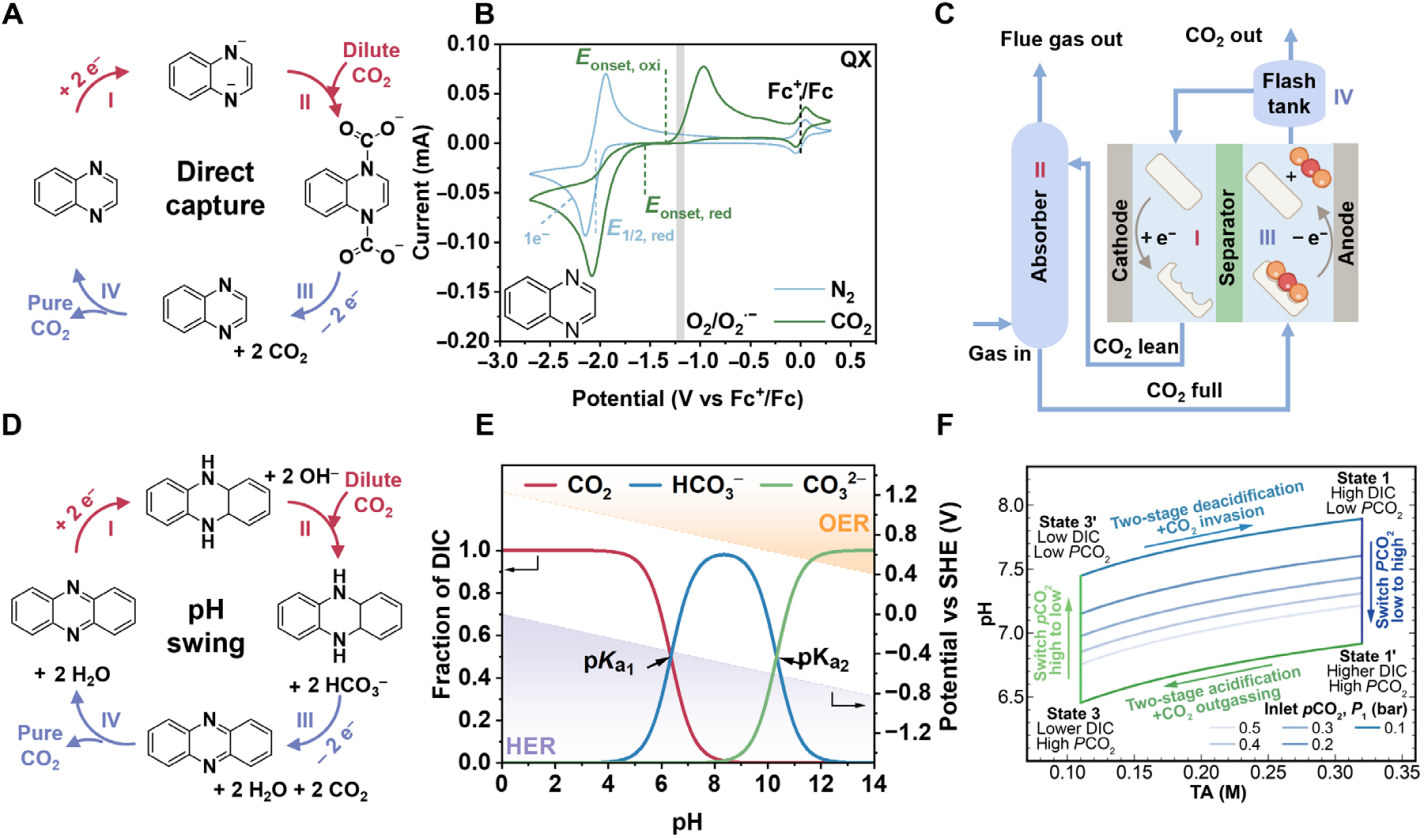
sp^2^-N–mediated CO_2_ capture. (**A**) Demonstration of the ″catch and release″ process in direct capture. (**B**) Cyclic voltammograms of representative sp^2^-N (QX) for EMCC, adapted with permission from Li *et al.* (*20*). (**C**) Process flow diagram for the electrochemical CO_2_ capture. (**D**) pH-swing CO_2_ capture. (**E**) The Bjerrum plot at 25°C shows the distribution of CO_2_ (red), HCO_3_^−^ (blue), and CO_3_^2−^ (green) across the pH range, with shaded regions indicating the potential for HER and OER window (1 to 14) (*9*, *143*). (**F**) pH versus total alkalinity (TA) in ideal pH-swing CO_2_ capture cycles. Arrows indicate experimental process directions across four key states: CO_2_ release with headspace pressure drop (state 1 → 1′), acidification and CO_2_ outgassing from DIC (state 1′ → 3), pressure recovery (state 3 → 3′), and two-stage electrochemical deacidification with CO_2_ invasion (state 3′ → 1), adapted with permission from Jin *et al.* (*44*).

For the direct CO_2_ capture methodology, sp^2^-N serves as a direct CO_2_ catcher without the incorporation of other competitors (Eqs. 5 to 7). Typical sp^2^-N structures that can be used for direct capture include bipyridine, azole, quinoxaline (QX), PhN, azo, etc. Here, we take QX as an example to illustrate capture process and underlying mechanism. To be specific, its nucleophilicity toward CO_2_ is enhanced by the uptake of two electrons under a reduced potential, promoting CO_2_ capture and formation of the QX(COO)_2_^2−^ adduct. Subsequently, under a reverse potential, QX(COO)_2_^2−^ loses two electrons and is oxidized to QX with the release of CO_2_ molecules (Fig. 3A) (*20*, *25*). Figure 3B shows cyclic voltammograms of QX under N_2_ and CO_2_, where current switches at different potentials highlight CO_2_ involvement in the redox process. Early studies used a variety of redox-active organic materials (ROMs), including quinones, pyridines, and disulfides, to demonstrate the reversibility of [ROMCO_2_]*_x_*^−^ formation in CO_2_ environments. This reversibility validated the feasibility of using ROMs for EMCC (*26*–*29*). However, they suffered from problems of difficult synthesis, low aqueous solubility, high oxygen sensitivity, and CO_2_ adduct irreversibility

$$\text{QX}+{\text{2e}}^{-}\to {\text{QX}}^{2-}$$ (5)

$${\text{QX}}^{2-}+{\text{2CO}}_{2}\to \text{QX}{{\left(\text{COO}\right)}_{2}}^{2-}$$ (6)

$$\text{QX}{{\left(\text{COO}\right)}_{2}}^{2-}-{\text{2e}}^{-}\to \text{QX}+{\text{2CO}}_{2}$$ (7)

The development of sp^2^-N compounds for direct CO_2_ capture has evolved from broad screening of potential candidates to precise fine-tuning of molecular structures and electrolytes. Research on this type of process began with a stable reduced *N*-propyl-4,4′-bipyridinium capable of capturing and releasing CO_2_ through the modulation of redox potentials (*27*). The reaction of 4,4′-bipyridine (Bpy) with CO_2_ at a rapid rate constant of 9.2 × 10^7^ M^−1^ s^−1^ reveals that the reduced sp^2^-N─CO_2_ interaction is also spontaneous. Its excellent stability and reversibility over 20 cycles provide preliminary evidence for the feasibility of this process (*30*). Under identical conditions, a range of sp^2^-N structures with varying Lewis’s basicity demonstrated that the interaction strength between CO_2_ and reduced sorbents is proportional to the local electron density at the nitrogen center. Notably, 4,4′-azopyridine (AzPy) with its extended conjugation currently emerged as the most effective compound for electrochemical CO_2_ capture. Figure 3C illustrates the overall process of sp^2^-N–mediated CO_2_ capture/release from a CO_2_ absorber to an electrochemical reactor. In this work, AzPy achieved nearly 100% CO_2_ capture from a 20% CO_2_ feed, maintaining 90% capture efficiency and more than 80% coulombic efficiency after 20 cycles while retaining its performance in the presence of 3% O_2_ (*20*). Reduced sp^2^-N exhibits pseudo-amine properties, enabling rapid CO_2_ capture through a near-identical capture pathway and efficient C─N bond cleavage with sorbent regeneration via redox potential tuning. However, electrophilic impurities (e.g., H^+^ and O_2_) necessitate nonaqueous systems. We propose that future efforts should probably focus on enhancing impurity resistance, optimizing the redox potential window, and improving capture reversibility for both fundamental studies and industrial applications.

### sp^2^-N–mediated pH-swing CO_2_ capture

In addition to serving as direct CO_2_ capture agents, certain sp^2^-N compounds tend to bind with protons (H^+^), forming protonated adducts that facilitate water dissociation during the electrochemical reduction process (Eqs. 8 to 10). This process induces solution pH shift and further electrochemically converts captured CO_2_ into aqueous carbonates (*6*, *31*). Figure 3D illustrates how these sp^2^-N compounds (exemplified by PhN) indirectly regulate CO_2_ capture via proton-coupled electrotransfer reactions, which induce a pH swing via oxidation and reduction processes. Figure 3E presents a Bjerrum plot that illustrates the carbonic acid equilibrium across a wide pH range, showing how the pH swing governs the distribution of dissolved inorganic carbon (DIC) (*32*). Below pH ~4, CO_2_ remains largely as gas, limiting its dissolving (capture). Between pH 4 and 12, it progressively converts to HCO_3_^−^ and CO_3_^2−^, and above pH 12, CO_3_^2−^ dominates. Thus, capture agents should operate within an optimal pH range (~6.5 to 8.0), distinct from traditional thermal scrubbing (~8 to 10) (*33*), with HCO_3_^−^ as the dominant dissolved CO_2_ species in this range. This enables reversible CO_2_ interaction while avoiding excessive acidification and excessive alkalization (which risks carbonate precipitation and reduced CO_2_ activity). As shown in Fig. 3F, after sp^2^-N compounds are reduced (state 3′ → 1) to sp^2^-NH_2_, the enriched hydroxyl ions (OH^−^) work as the authentic carbon catchers, trapping CO_2_ and forming DIC species (HCO_3_^−^/CO_3_^2−^; state 1 → 1′) (*34*–*37*). Upon oxidation (state 1′ → 3), the PhNH_2_ adducts lose electrons, regenerating PhN and releasing protons into the electrolyte, consequently lowering the solution pH and converting DIC species back to CO_2_, thereby achieving CO_2_ enrichment (state 3 → 3′) (*28*). Earlier studies suggested that ideal proton-coupled electrotransfer redox molecules should have a redox potential situated between the hydrogen evolution reaction (HER) and the oxygen evolution reaction (OER) across a specific pH range, along with high reversibility, a favorable proton-to-electron transfer ratio, and resistance to both electrochemical degradation and oxygen exposure (*37*). Therefore, from an electrochemical potential perspective alone, this process offers certain advantages over other CO_2_ electrochemical capture approaches that rely on the oxygen reduction reaction (ORR)/OER couple, which also use OH^−^ generation during reduction (*38*). Good representatives of sp^2^-N compounds for pH-swing capture include quinoline, phenols, tyrosine, and PhN (*25*, *39*–*41*). Specifically, we summarized the electrochemically mediated CO_2_ capture process in Table 2

$$\text{PhN}+{\text{2H}}_{2}O+{\text{2e}}^{-}\to {\text{PhNH}}_{2}+{\text{2OH}}^{-}$$ (8)

$${\text{2OH}}^{-}+{\text{2CO}}_{2}\to {{\text{2HCO}}_{3}}^{-}$$ (9)

$${\text{PhNH}}_{2}+{{\text{2HCO}}_{3}}^{-}-{\text{2e}}^{-}\to \text{PhN}+{\text{2H}}_{2}O+{\text{2CO}}_{2}$$ (10)

**Table 2.** Summary of sp^2^/sp^3^-N sorbent agents for electrochemical carbon capture processes.

sp^2^/sp^3^ structures	Capture agent	Capture mechanism	Reduction potential^*^	Separation energy (kJ mol^−1^)	Key features	Ref.
sp^2^-N	*N*-Propyl-4,4′-bipyridinium (Prbipy^+^)	Direct	–	–	–	(*27*)
	4, 4′-Bipyridine (Bpy)	Direct	–	–	–	(*30*)
	*N*-Methyl-4,4′-bipyridinium (Mebipy^+^)	Direct	−1.65 V vs Ag/Ag^+^	–	–	(*149*)
	2,1,3-Benzothiadiazole (BTZ)	Direct	−1.8 V vs Ag/AgNO_3_	–	Irreversible reaction with CO_2_	(*50*)
	Polybenzothiadiazole (PBTZ)	Direct	−1.9 V vs Ag/AgNO_3_	–	Redox-active polymer	(*50*)
	4,4′-Bipyridine (Bpy)	Direct	−1.67 V vs Fc^+^/Fc	–	–	(*20*)
	Quinoxaline (QX)	Direct	−1.548 V vs Fc^+^/Fc	–	Study the influence of introducing EWGs (Cl, CF_3_, and CN)	(*20*)
	Phenazine (PhN)	Direct	−1.41 V vs Fc^+^/Fc	–	Study the influence of introducing EWGs (OH and CF_3_)	(*20*)
	2,1,3-Benzothiadiazol (BNSN)	Direct	−1.691 V vs Fc^+^/Fc	–	Study the influence of introducing EWGs (F and CF_3_)	(*20*)
	Azobenzene (AzB)	Direct	−0.933 V vs Fc^+^/Fc	–	–	(*20*)
	4,4′-Azopyridine (AzPy)	Direct	−0.933 V vs Fc^+^/Fc	120 (18.5% CO_2_)	Highest release/capture efficiency among compounds for direct capture	(*20*)
	1-Aminopyridinium (1-AP)	pH swing	−0.45 V vs Ag/AgCl	101 (DAC^†^)	1-AP nitrate is a redox-active amine absorbent	(*35*)
	2,2′-(Phenazine-1,8-diyl) bis(ethane-1-sulfonate) (1,8-ESP)	pH swing	−0.3 V vs SHE @ pH 7	55 (10% CO_2_)	High aqueous solubility (>1.35 M) and CO_2_ capture capacity (vol): 1.4 M CO_2_	(*7*)
	Sodium (3,3′-(phenazine-2,3-Diylbis(oxy)) bis(propane-1-sulfonate)) (DSPZ)	pH swing	−0.18 V vs SHE @ pH 9	121 (DAC), 79.4 (9.1% CO_2_)	Introduce electrorebalancing method to restore the initial composition of the electrolytes.	(*37*, *44*)
	7,8-Dihydroxyphenazine-2-sulfonic acid (DHPS)	pH swing	−0.56 V vs Ag/AgCl @ pH 7	21.6 (15% CO_2_)	High proton transfer number per electron (3H^+^/2e^−^).	(*25*)
	2,3-Bis(2,5,8,11-tetraoxatridecan-13-yloxy) phenazine (*o*-BTEP)	pH swing	–	48.4 (15% CO_2_)	Mutual solubility in deionized water.	(*25*)
	Riboflavin 5′-monophosphate sodium salt hydrate (FMN)	pH swing	−0.5 V vs Ag/AgCl @ pH 8	9.8 (15% CO_2_, 60°C)	The capture agent FMN is a biological proton carrier.	(*45*)
	Neutral red (NR)	pH swing	−0.61 V vs Ag/AgCl @ pH 6	35 (15% CO_2_), 65 (DAC)	Use nicotinamide as a hydrotropic solubilizing agent.	(*34*)
sp^3^-N	1,2-Dimethylimidazole (DMID)	EMAR	–	65 (15% CO_2_)	The Cu(I)/Cu redox couple minimizes energy consumption.	(*68*)
	Ethylenediamine (EDA)	EMAR	*E*^0^ (Cu^2+^/Cu)	50 (15% CO_2_)	The pioneering work on the concept of EMAR.	(*4*, *65*)
	50/50 mixture of EDA and aminoethylethanolamin (AEEA)	EMAR	*E*^0^ (Cu^2+^/Cu)	35 (15% CO_2_)	The overall process efficiency was enhanced through the utilization of an amine blend.	(*53*)
	Monoethanolamine (MEA)	EMAR	*E*^0^ (Cu^2+^/Cu)	67.76 (N.A.)	A current density of 150 A/m^2^ was achieved using modular flow-through electrolysis cells.	(*55*, *150*)

*Reduction potential is the onset potential obtained under CO_2_ environment.

†DAC refers to direct air capture.

Here, we briefly outline several representative pH-swing processes. For instance, 1-aminopyridinium (1-AP), which is initially inactive toward CO_2_ in a neutral aqueous solution, becomes reactive upon electroreduction. Its unique NH_2_-pyridinic structure allows for the capture of two CO_2_ molecules per electron transferred (*42*). This process achieved an energy cost of 101 kJ mol^−1^ CO_2_ and an electron utilization efficiency of 0.78 for direct air capture (DAC) (*24*, *35*). For another, PhN and its derivatives have emerged as effective proton acceptors for pH-swing CO_2_ capture because of their electron-rich heterocyclic π-systems, dual nitrogen atoms that provide a high proton capacity (2 mol H^+^ per mol PhN), and excellent redox reversibility in aqueous systems (*25*, *37*). These compounds offered straightforward synthesis, customizable properties, high stability, and low equilibrium overpotential [−0.34 V versus standard hydrogen electrode (SHE) at pH 8] (*37*, *43*). Depending on this field advancement, PhN derivatives, as a proof of concept, also demonstrated excellent high-capacity and high-stability performance in capturing diluted CO_2_ such as from simulated flue gas and air-source CO_2_ (*25*, *44*). By designing and tuning functional groups and their positions, energy consumption decreased substantially from 61.3 kJ mol^−1^ CO_2_ [sodium (3,3′-(phenazine-2,3-diylbis(oxy))bis(propane-1-sulfonate)) (DSPZ)] to 35 kJ mol^−1^ CO_2_ (neutral red) for flue gas capture and from 121 to 65 kJ mol^−1^ CO_2_ for DAC (*34*). Subsequently, Pang *et al.* (*7*) successfully coupled CO_2_ capture with electricity storage using another PhN derivative, 2,2′-(phenazine-1,8-diyl) bis(ethane-1-sulfonate) (1,8-ESP). During the capture step, the system charged and subsequently delivered electrical energy with the release of pure CO_2_ gas. In addition to these typical sp^2^-N compounds, the research community has highlighted flavin derivatives, such as riboflavin 5′-monophosphate sodium salt hydrate (FMN), which feature unique heterocyclic structures and exhibit impressive biological proton-carrying functionality. Inspired by their structural features, this work achieved a high faradic efficiency (FE) (94.3%) and low energy consumption (9.8 kJ mol^−1^ CO_2_) (*45*). Indirect CO_2_ capture via pH swing resembles tertiary amine systems, forming (bi)carbonate species through acid-base equilibria, while direct capture is akin to primary amine chemistry (direct N─C bond formation) with faster kinetics. Although pH swing is slower, it enables aqueous operation and offers lower thermodynamic energy requirements. The oxygen and moisture tolerance of sp^2^-N sorbents has been demonstrated in circulation-mode studies, supporting their potential in nonthermal CO_2_ enrichment. However, the influence of acidic impurities (e.g., SO*_x_* and NO*_x_*) warrants further evaluation. These features make sp^2^-N sorbents strong candidates for scalable, energy-efficient pH-swing CO_2_ capture. These advancements position sp^2^*-*N sorbents as promising candidates for scalable and energy-efficient pH-swing CO_2_ capture technologies.

### Strategies for enhancing sp^2^-N–mediated CO_2_ capture

**Fig. 4.**
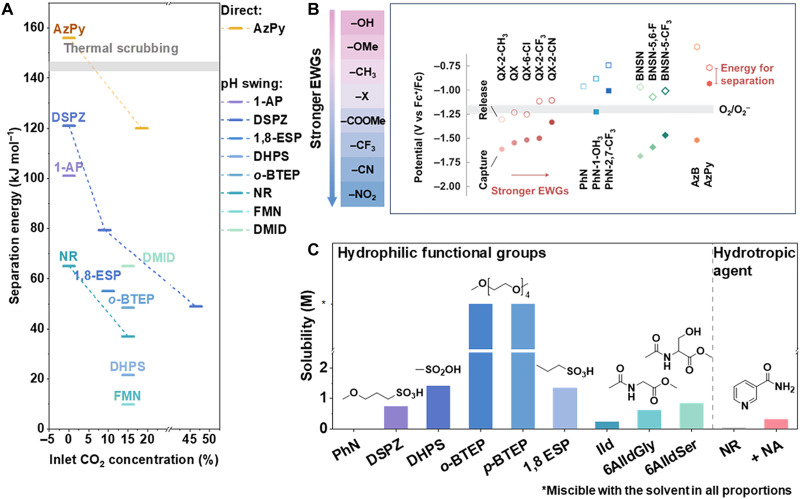
Strategies for enhancing sp^2^-N–mediated CO_2_ capture. (**A**) Energy requirement for EMCC with varied conditions (CO_2_ concentrations, capture agents, etc.). The middle line represents the reference range for traditional thermal amine scrubbing for CO_2_ separation, with an energy requirement of 141 to 242 kJ mol^−1^ CO_2_ (*7*, *20*, *25*, *34*, *35*, *44*, *45*, *144*). (**B**) Onset potentials for CO_2_ capture (filled symbols) and release (open symbols) for various sorbents, arranged by increasing EWG strength. Adapted with permission from Li *et al.* (*20*). (**C**) Solubility of sp^2^-N compounds with different functional groups (*7*, *21*, *24*, *25*, *34*, *37*, *44*, *145*).

Tremendous efforts have been dedicated to the sp^2^-N–mediated electrochemical CO_2_ capture field, leading to the development of various molecular structures and diverse reaction pathways designed to enhance capture efficiency. Figure 4A outlines the energy requirements for the recent electrochemical CO_2_ capture process at varying CO_2_ concentrations. To advance this field, we propose several strategies to optimize the overall CO_2_ enrichment process, including electronic structure modulation, impurity tolerance and recovery strategies, solubility improvements, and so forth.

#### Electronic modulation

At first, we summarize the electronic modulation strategy to adjust redox potentials and CO_2_ binding energies of these sorbents via the selection and design of different sp^2^-N molecules. Figure 4B compares various sorbents of sp^2^-N centers, showing that lower basicity correlates with a more positive half-wave potential (*E*_1/2_) and the binding energy follows the order 2,1,3-benzothiadiazol (BNSN) > QX > azobenzene (AzB) > PhN > AzPy (*20*). In general, electron-rich nitrogen centers with higher basicity require more energy to accept electrons, leading to lower *E*_1/2_. Conversely, other sp^2^-N compounds, such as PhN and azo compounds, which exhibit weaker affinity for CO_2_, have longer N─C bonds. These compounds can enhance system efficiency by promoting reaction reversibility through an anodic shift in redox potential. Notably, this anodic shift prevents reduced sorbents from being oxidized by O_2_ (from O_2_ to O_2_^−^ at −1.2 V versus Fc^+^/Fc) in nonprotic electrolytes (*46*, *47*). It is widely accepted that introducing stronger electron-withdrawing groups (EWGs) can tune the electronic density of nitrogen atoms and enhance interactions with CO_2_. Inspired by that, specific substituents such as ─F, ─Br, ─COOCH_3_, and ─NO_2_ were incorporated to sp^2^-N structures to progressively shift the redox potential to more anodic values (*21*). They claimed that the extent of this shift depends on the number and positioning of the substituents as placing these EWGs in appropriate positions would induce dipole-dipole repulsion or steric hindrance, resulting in a more reversible second redox process (*20*, *21*, *29*). Apart from the above aspects, the capture capabilities of these capture agents like anthraquinone derivatives can be enhanced substantially by introducing additional N-containing groups to modify their redox properties. For instance, the cyclic voltammetry of 1,4-NH_2_-anthraquinone under CO_2_-saturated conditions revealed merged reduction peaks, showing stronger interactions with CO_2_ than that of anthraquinone (*48*). This improvement can be attributed to the increased CO_2_ binding affinity of the amino groups, which induced greater electrochemical shifts.

#### Impurity tolerance and recovery strategies

Redox-tunable sp^2^-N frameworks enable efficient electrochemical CO_2_ capture but are highly susceptible to flue gas impurities like O_2_, H_2_O, and sulfur because of the elevated reactivity of their reduced states (*9*). In current studies, direct capture processes have quantified the effects of O_2_ and H_2_O, while investigations into indirect capture primarily focus on O_2_. In systems cycling between high-potential “release” and low-potential “capture” states, the reduced sp^2^-N moiety readily undergoes electron transfer with O_2_, initiating the ORR (Eq. 11) (*49*). At the molecular level, designing capture agents with reduction potentials beyond the oxygen reduction threshold—such as Azpy—effectively mitigates O_2_ interference, maintaining more than 85% capacity utilization under simulated flue gas containing 15% CO_2_ and 5% O_2_ (*20*). In another strategy, electrochemical rebalancing (Eqs. 12 to 14) was used under a constant current. As a result of the rebalancing reactions, the overall cell voltage dropped to negative values. Meanwhile, the pH on the cathode side decreased, which raised the potential of the anodic half-reaction and further lowered the overall cell voltage. The attainment of a constant state indicated the completion of the electrochemical rebalancing process. After the process, the oxidized sorbents were reduced, and consequently, their CO_2_ capture capability was restored. Another approach to enhancing the stability of these capture agents is incorporating sp^2^-N monomers into polymers to functionalize electrodes with electroswing adsorption properties (*50*). The polymerized N-rich polymers, polyanthraquinone and polybenzothiadiazole (PBTZ), exhibited anodic shifts in *E*_red_ during cyclic voltammetry under a CO_2_ environment compared to their monomeric counterparts. SO*_x_* and NO*_x_* in flue gas are also nonnegligible impurities that can reduce CO_2_ capture efficiency. However, to date, no electrochemical CO_2_ capture studies based on sp^2^-N compounds have investigated this aspect. Therefore, we address this issue in the “sp^3^-N–mediated reactive CO_2_ capture” section as part of our review of both sp^2^-N and sp^3^-N systems.

Oxygen influence

$$0.{\text{5O}}_{2}+{\text{DSPZH}}_{2}\to \text{DSPZ}+H_{2}O$$ (11)

Electrochemical rebalancing

$$\text{Anodic}:{\text{2OH}}^{-}\to 0.{\text{5O}}_{2}+H_{2}O+{\text{2e}}^{-}$$ (12)

$$\text{Cathodic}:{\left[K^{+}\right]}_{3}{\left[\text{Fe}{\left(\text{CN}\right)}_{6}\right]}^{3-}+e^{-}\to {\left[K^{+}\right]}_{4}{\left[\text{Fe}{\left(\text{CN}\right)}_{6}\right]}^{4-}$$ (13)

$${\text{CO}}_{2}\text{evolution}:{{\text{HCO}}_{3}}^{-}\to {\text{CO}}_{2}+{\text{OH}}^{-}$$ (14)

#### Improving solubility

Another important parameter that affects the capture efficiency should be indexed to the solubility of sp^2^-N compounds. However, N-containing heterocycles (sp^2^-N) typically exhibit low solubility because of the hydrophobic and nonpolar nature of their aromatic rings, unless other polar groups are present—unlike sp^3^-N amines. Hence, sp^2^-N with good solubility over a wide pH range is essential for ensuring homogenization and preventing precipitation during pH-swing CO_2_ capture. Otherwise, their CO_2_ capture capacity per unit volume is notably limited. Earlier works indicated that incorporating hydrophilic functional groups, such as sulfonic, carboxylic, or hydroxyl groups, enhances water solubility by facilitating stronger interactions with water molecules. The position and electronic environment of these groups within the molecule further influence the extent of this enhancement, as shown in Fig. 4C. On the basis of this perspective, substituents such as sulfonyl hydroxide [─SO_2_OH, 7,8-dihydroxyphenazine-2-sulfonic acid (DHPS)], ethane-1-sulfonate (─C_2_H_4_SO_3_H, ESP), and 3-sulfopropoxy (─O─C_3_H_6_SO_3_H, DSPZ) were introduced onto the aromatic ring, resulting in notable solubility improvement (*25*, *51*). As for the effect of substituted positions, it affects the hydrophilic property of the sp^2^-N compound because of their distinct molecular conformation and electronic effects. To be specific, the spatial orientation of hydrophilic groups affects their interaction with water, while the nitrogen atom can influence the hydrophilic group’s polarity via electronic donation or withdrawal, affecting the overall solubility. On this basis, solubility improvements were achieved for 1,8-ESP, reaching 1.4 M in 1 M KCl, 1.6 M in 1 M H_2_SO_4_, and up to 2.06 M in 2 M KOH, surpassing the symmetrically substituted 2,7-ESP and 1,6-ESP (*37*). An alternative approach, without altering the molecular structure, involves using hydrotropic agents to enhance solubility by reducing interfacial tension, improving solvation, or forming micellar structures. Seo and Hatton (*34*) incorporated 1 M nicotinamide into 0.5 M KCl and increased the solubility of the PhN derivative neutral red from 46 to 306 mM. The use of hydrotropic agents, rather than synthetic modifications, preserved the intrinsic robustness of neutral red systems against oxygen, enabling PhN-based systems to achieve direct CO_2_ capture from air with low energy consumption (*34*).

#### Electrolyte regulation

As the final strategy, researchers aimed to improve CO_2_ capture performance by introducing salts or additives into the electrolyte, which actively modulated the chemical environment and influence redox behavior. When sp^2^-N is used as a direct sorbent, added cations form coordination bonds with negatively charged sorbent-CO_2_ adducts, and cation size plays a critical role. In AzPy solutions, smaller cations like Na^+^ exhibit stronger coordination than larger ones like tetrabutylammonium cation (TBA^+^), resulting in a 300 mV higher adduct oxidation potential, effectively mitigating the impact of O_2_ (O_2_/O_2_·^−^) (*20*, *21*). In a recent sp^2^-N–based CO_2_ capture study, efforts to identify suitable nonaqueous solvents have aimed to address volatility losses, oxygen sensitivity, and high regeneration energy. Low-volatility alcohols or nonnucleophilic superbases have been explored as water substitutes. In such systems, rather than generating hydroxide as in conventional pH-swing processes, sp^2^-N species like AzB act as Brønsted bases to deprotonate alcohols, enabling the conversion of CO_2_ into alkyl carbonates (Eqs. 15 and 16). Theoretically, all species involved in the cyclic process are air-stable, providing a mechanistic solution to address the O_2_ sensitivity issue in EMCC systems (*52*)

$$\text{AzB}+\text{2ROH}+{\text{2e}}^{-}\to {\text{AzBH}}_{2}+{\text{2RO}}^{-}$$ (15)

$${\text{2RO}}^{-}+{\text{2CO}}_{2}\to {{\text{2ROCO}}_{2}}^{-}$$ (16)

Insights from electromediated amine regeneration (EMAR) suggest that introducing a surfactant, such as SDS, at subcritical micelle concentrations can notably enhance the CO_2_ release process. By forming a bilayer on the positively charged anode, the surfactant reduces electrode instability, minimizes gas bubble formation, and lowers the cell potential required for CO_2_ desorption, thereby improving the overall system efficiency (*53*).

### System and operational optimization

Similar to traditional thermal amine stripping, the most common electrochemical CO_2_ capture configuration functions as a continuous capture-release process, where CO_2_ absorption and desorption take place in separate compartments. Numerous studies on direct CO_2_ capture and pH-swing CO_2_ capture use setups that integrate a capture column with an electrochemical cell (*54*–*56*). These systems often use sp^2^-N compounds for reversible CO_2_ binding and release. Under reduction conditions, sp^2^-N compounds are reduced so as to capture CO_2_ in the absorption column until saturation. Subsequently, CO_2_-rich solutions are transferred to the anode chamber where oxidation happens with the release of CO_2_. The CO_2_-lean solutions are then returned to the cathode chamber to complete the cycle. While this single-pass process simplifies system design and operation, adjusting parameters such as current density, flow rate, and FE presents a challenge. Therefore, ineffective control can result in incomplete desorption, leading to reduced CO_2_ absorption and overall cell capacity over time. Advanced valving and control systems may be necessary to maintain stability and consistency. In an alternative system design, the cathode and anode are completely separated by a membrane, preventing crossover between the catholyte and the anolyte. Like the battery charging and discharging process, sp^2^-N compounds are reduced during the charging phase, accepting protons to increase the solution pH or directly generating radicals. This pH increase raises the solution’s alkalinity, allowing CO_2_-rich gas to be fed into the electrolyte, where it forms carbonates or CO_2_ adducts. Once saturated with CO_2_, the cell polarity is reversed in the discharge phase. Without transferring to the opposite chamber, the CO_2_-loaded solution undergoes oxidation on the same side, which either releases protons to lower the pH or directly releases pure CO_2_. To complete the electrical circuit, a redox reaction is typically coupled at the opposite electrode. Reported reactions include ferrocyanide (*7*, *37*), derivatized ferrocene (*20*, *57*), or the same redox couple. Because both absorption and desorption occur in the same chamber, regulating capture rate, flow rate, and current density is more straightforward. However, polarity transitions can cause system fluctuations, potentially disrupting smooth operation (*49*). In short, sp^2^-N compounds enable CO_2_ capture primarily through electrochemically controlled redox tuning at N sites or via nucleophilic interactions that donate electron density into the empty π* orbitals of the CO_2_ carbon center. However, the redox activity remains largely localized on the sp^2^-N framework, and the captured CO_2_ molecule typically remains in its oxidized form without undergoing structural upgrading. In the following section, we explore the emerging role of sp^3^-N compounds, which can spontaneously form adducts with CO_2_ through strong nucleophilic interactions. These systems enable more dynamic electrochemical behaviors: either through metal-cantered redox processes that modulate CO_2_ affinity or via direct participation of CO_2_ in redox-driven transformations that facilitate amine regeneration and pave the way for CO_2_ upgrading into value-added products.

## SP^3^-N–MEDIATED ELECTROCHEMICAL CO_2_ CAPTURE AND REACTIVE CO_2_ CAPTURE

**Fig. 5.**
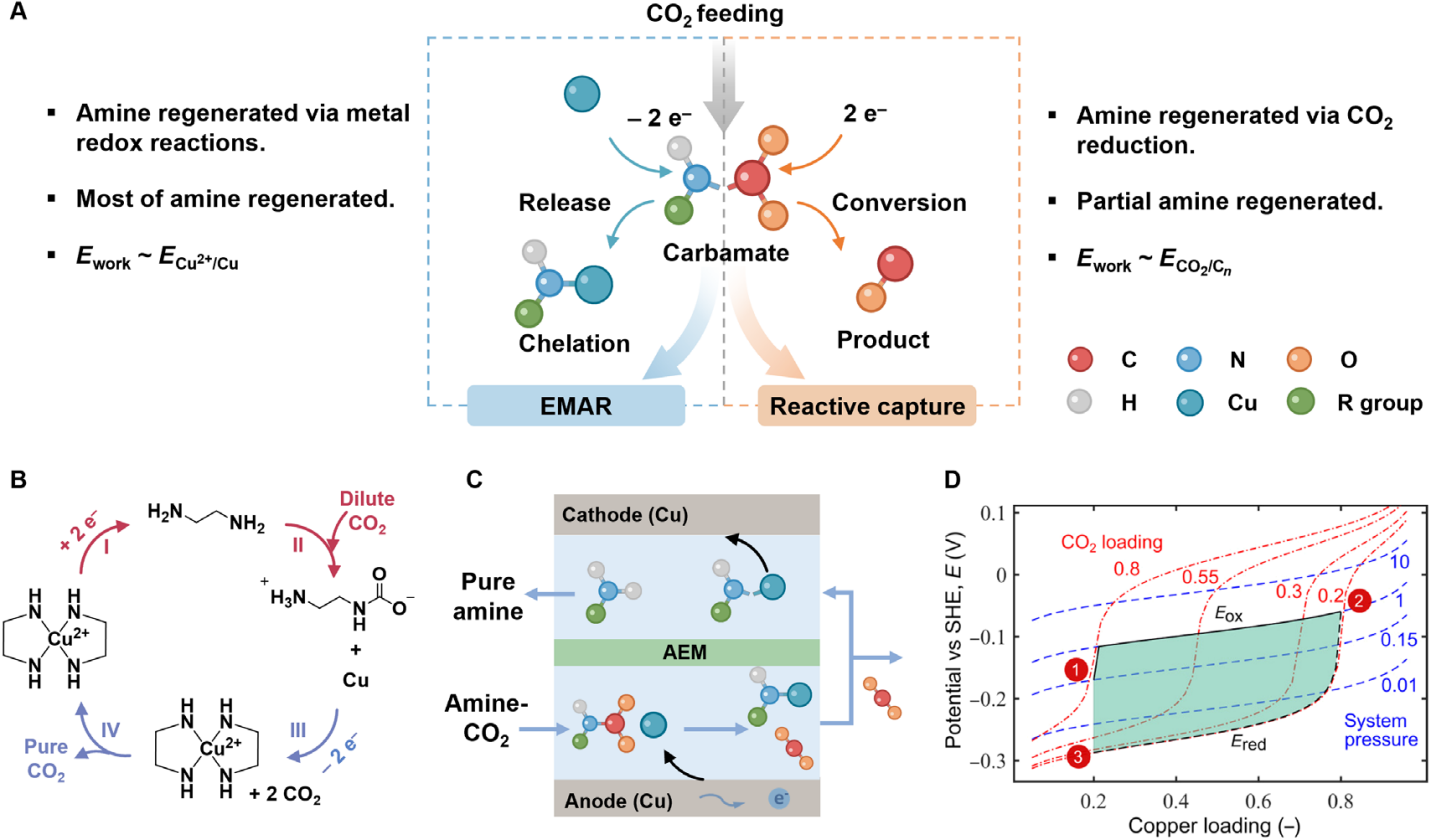
sp^3^-N–mediated CO_2_ capture. (**A**) Illustration of amine regeneration via two processes: EMAR (left) and reactive capture (right). (**B**) Demonstration of the EMAR process. (**C**) Process flow diagram for the EMAR process. (**D**) Thermodynamic pathway of the EDA-H_2_O-CO_2_ system. The shaded area represents the electrochemical working window between the Cu oxidation (*E*_ox_) and reduction (*E*_red_) potentials as a function of Cu loading. Contours show CO_2_ loading (red, dashed lines) and system pressure (blue, dashed). State 1: The CO_2_-rich EDA solution reacts with electrochemically generated Cu^2+^, releasing CO_2_. State 2: The lean solution is regenerated at the cathode by Cu^2+^ reduction. State 3: Lean EDA returns to the absorber for CO_2_ capture. Adapted with permission from Wang *et al.* (*59*).

As discussed before, sp^3^-N compounds (amines) interact with CO_2_ to form carbamates or (bi)carbonates to achieve CO_2_ adsorption. Subsequently, high-purity CO_2_ is released via C─N/C─O bond cleavage through the thermal regeneration of amine-based materials. In comparison, sp^3^-N compounds provide a dual electrochemical function: They enable CO_2_ capture and release via electrochemical processes or facilitate its conversion into high-value products (Fig. 5A). The first is known as the EMAR process, where CO_2_-captured amines preferentially coordinate with metal ions to achieve C─N bond cleavage and high-purity CO_2_ release by shifting applied voltage. Distinct from electrochemical sp^2^-N systems above, where redox activity occurs on the N center, EMAR processes present redox reactions on the metal electrode, maintaining the amine in a constant oxidation state throughout. The second approach is the recently emerging reactive CO_2_ capture, also known as integrated CO_2_ capture and conversion, which uses electroreduction to supply additional electrons to carbon orbitals, facilitating C─N bond cleavage and CO_2_ mineralization. Consistent with traditional amine scrubbing processes, EMAR and reactive CO_2_ capture require sp^3^-N compounds with a fast CO_2_ capture rate, high CO_2_ capture capacity, and low heat of regeneration to enable efficient and repeatable “capture and release” cycles. To advance this burgeoning technology, this section presents the mediation of sp^3^-N structures in the fields of electrochemical CO_2_ capture and reactive CO_2_ capture. In these two configurations, the involvement of sp^3^-N compounds to better promote the carbon capture efficiency and CO_2_ conversion from the aspects of amine sorbents, catalysts, working electrolytes, and separators and their fundamental interaction rationale for bringing such improvements are discussed.

### sp^3^-N–mediated electrochemical amine regeneration for CO_2_ capture

EMAR is based on the principle that the coordination bonds formed between metal ions and amines exhibit extremely higher formation rates and thermodynamic stabilities compared to N─CO_2_ bonds. As a result, electrochemical mediation of the metal^+^/metal redox cycling can effectively regulate amine coordination (*58*). In this setup, aqueous amine solutions serve as the working electrolyte, while transition metals or their complexes, which can coordinate with amine electrolytes under an applied potential, act as the electrode (Fig. 5, B and C). Compared with conventional amine scrubbing, EMAR has benefits of high energy efficiency and system conciseness (*55*). Earlier EMAR studies screened monoamines, amino acids, and polyamines, selecting amines with at least two N sites for their Cu^2+^ chelation ability and prevention of salt precipitation. Moreover, ethylenediamine (EDA) had been further selected owing to its high CO_2_ capacity, low cost, fast kinetics, and low viscosity, even in CO_2_-saturated solutions. The amine (EDA)–related ions under different Cu loadings are shown in (*54*). Figure 5D illustrates the system’s behavior under varying potentials, with three labeled states representing distinct solvent conditions: (i) CO_2_-rich, Cu-lean; (ii) CO_2_-lean, Cu-rich; and (iii) CO_2_-lean, Cu-lean, corresponding to reactions (Eqs. 17 to 19, respectively)

$$\text{2EDA}─{\text{CO}}_{2}+{\text{Cu}}^{0}-{\text{2e}}^{–}\to {\left[\text{Cu}{\left(\text{EDA}\right)}_{2}\right]}^{2+}+{\text{2CO}}_{2}$$ (17)

$${\left[\text{Cu}{\left(\text{EDA}\right)}_{2}\right]}^{2+}+{\text{2e}}^{-}\to \text{Cu}+\text{2EDA}$$ (18)

$$\text{2EDA}+{\text{2CO}}_{2}\to \text{2EDA}─{\text{CO}}_{2}$$ (19)

The introduction of Cu^2+^ ions into a CO_2_-rich EDA solution (state 1 → 2) electrochemically triggers CO_2_ desorption because the chelation reaction between EDA and Cu^2+^ is kinetically faster than the adsorption of CO_2_. During reduction (state 2 → 3), Cu^2+^ ions are plated back onto the electrode, regenerating the capture solution. State 2 → 3 is considered analogous to the traditional amine scrubbing process, where lean EDA captures CO_2_ from a dilute source in the absorption column. The blue lines represent constant CO_2_ partial pressure under different Cu loadings, highlighting the competition between CO_2_ and Cu^2+^ with EDA and showing that electrochemical regeneration enables CO_2_ release even at high pressures (*53*, *59*, *60*). Building on this concept, recent work in the EMAR system achieved an energy consumption of ~40 kJ mol^−1^ CO_2_ for electricity and ~80 kJ mol^−1^ CO_2_ for heat, notably lower than thermal regeneration, with amine regeneration ranging from 0.12 to 0.62 mol CO_2_ mol^−1^ amine (*61*–*63*). This strategy was first proposed by Hatton and colleagues (*64*, *65*), with early fundamental work focusing on screening amine sorbents and electrodes (EDA as the sorbent and cupric ions as the redox mediator), as well as developing operating procedures and optimizing conditions (*4*). Through thermodynamic and engineering advancements, a pilot plant was developed to capture 3.6 million tonnes of CO_2_ annually from a 550-MW coal-fired power plant (*59*, *66*). To mitigate the high-vapor-pressure operation and reduce EDA losses during the EMAR process, the amine blend strategy of EDA with aminoethylethanolamine (AEEA) was introduced as a CO_2_ cocapture agent under low-vapor-pressure conditions (*53*). As a result, amine loss was minimized to just 3% after 100 hours of continuous operation, while energy consumption was 10% lower in the EDA-AEEA (50/50) composition compared to the 100% EDA system. The benchmark amine monoethanolamine (MEA) demonstrated effective performance in the EMAR process, with bench-scale electrolysis flow cells featuring 20 electrodes achieving a desorption current of 5 A (~150 A m^−2^) at a cell voltage below 1 V (*55*). Recent work has focused on fine-tuning the process, with several key improvements enhancing the overall efficiency: incorporating low-vapor-pressure AEEA to create amine blends (*53*), adding inorganic salts like NaCl to facilitate metal dissolution and CO_2_ desorption (*67*), and using a Cu(I)/Cu redox pair with a smaller potential difference (*12*, *68*). In the following work, the specific applied potential of 1.0 to 1.5 V was recommended for improved CO_2_ desorption and reduced energy use (*54*). These findings guide process optimization and mark a key step toward more efficient EMAR technologies for CO_2_ capture and utilization.

To bridge the gap between bench-scale testing and industrial application, electrochemical CO_2_ capture should adapt to industrial gas streams such as flue gas that generally contains 12 to 15% CO_2_, 70 to 75% N_2_, 3 to 8% O_2_, and trace levels of SO*_x_* (e.g., 120 to 250 ppm of SO_2_) and NO*_x_* (150 to 250 ppm of NO) and to direct air, which contains ~0.04% CO_2_, ~21% O_2_, and ~78% N_2_ (*69*). However, impurities such as SO*_x_*, NO*_x_*, and O_2_ raise notable concerns regarding the long-term stability of sp^3^-N and reduced sp^2^-N compounds (*70*). A previous study has shown that key diamines in EMAR, such as EDA, primarily degrade via carbamate formation followed by cyclization into imidazolidinones or via nucleophilic attack by free amines forming urea (*71*). In several case studies, SO*_x_* have been shown to cause irreversible amine degradation through the formation of ammonium sulfate salts and organosulfur compounds such as thioglycolic acid (*72*, *73*). Similarly, NO*_x_* is known to react with secondary amines to form nitrosamines and nitramines, which are highly irreversible degradation products that cannot be removed by thermal regeneration (*74*, *75*). In addition, the intrinsic redox activity of SO*_x_* and NO*_x_* species, such as SO_2_/S (*E*^0^ ≈ 0.45 V), NO/NH_3_ (*E*^0^ ≈ 0.75 V), and NO_2_/NH_3_ (*E*^0^ ≈ 0.83 V) versus reversible hydrogen electrode (RHE), may lead to undesirable electrochemical side reactions that compromise system selectivity and FE (*76*, *77*). Furthermore, both impurities may form surface deposits on electrodes, resulting in catalyst poisoning and electrode deactivation (*77*). These acidic gases can react with Cu^2+^ ions in the electrolyte to form insoluble copper sulfides, leading to electrode degradation and loss of electrochemical activity over time (*78*). Thus, to enhance stability, alternative diamines with modified structures—such as the cyclic diamine piperazine (PZ) or hydroxyl-containing *N*,*N*′-bis(2-hydroxyethyl)ethylenediamine—may suppress degradation pathways and improve the system’s long-term performance (*79*). Aside from selecting more robust and specialized CO_2_ capture agents, several general strategies can be used to enhance the tolerability and stability of electrochemical capture under industrial conditions. Because of its acidic nature, wet flue gas desulfurization using limestone, lime, or seawater converts SO*_x_* into solid byproducts and removes more than 90% of SO_2_ before further process. Because of the complex composition of NO*_x_* and the extremely low solubility of certain species such as NO, conventional scrubbing methods are often ineffective. Current strategies include oxidative conversion of NO to more soluble NO_2_ followed by wet scrubbing (>90%) (*80*) or the complete reduction of NO*_x_* to inert N_2_ through catalytic (90 to 95%) or electrochemical pathways (*81*). To enable practical deployment, future electrochemical CO_2_ capture efforts must better reflect real flue gas compositions and thoroughly evaluate degradation pathways induced by impurities.

### sp^3^-N–mediated reactive CO_2_ capture

**Fig. 6.**
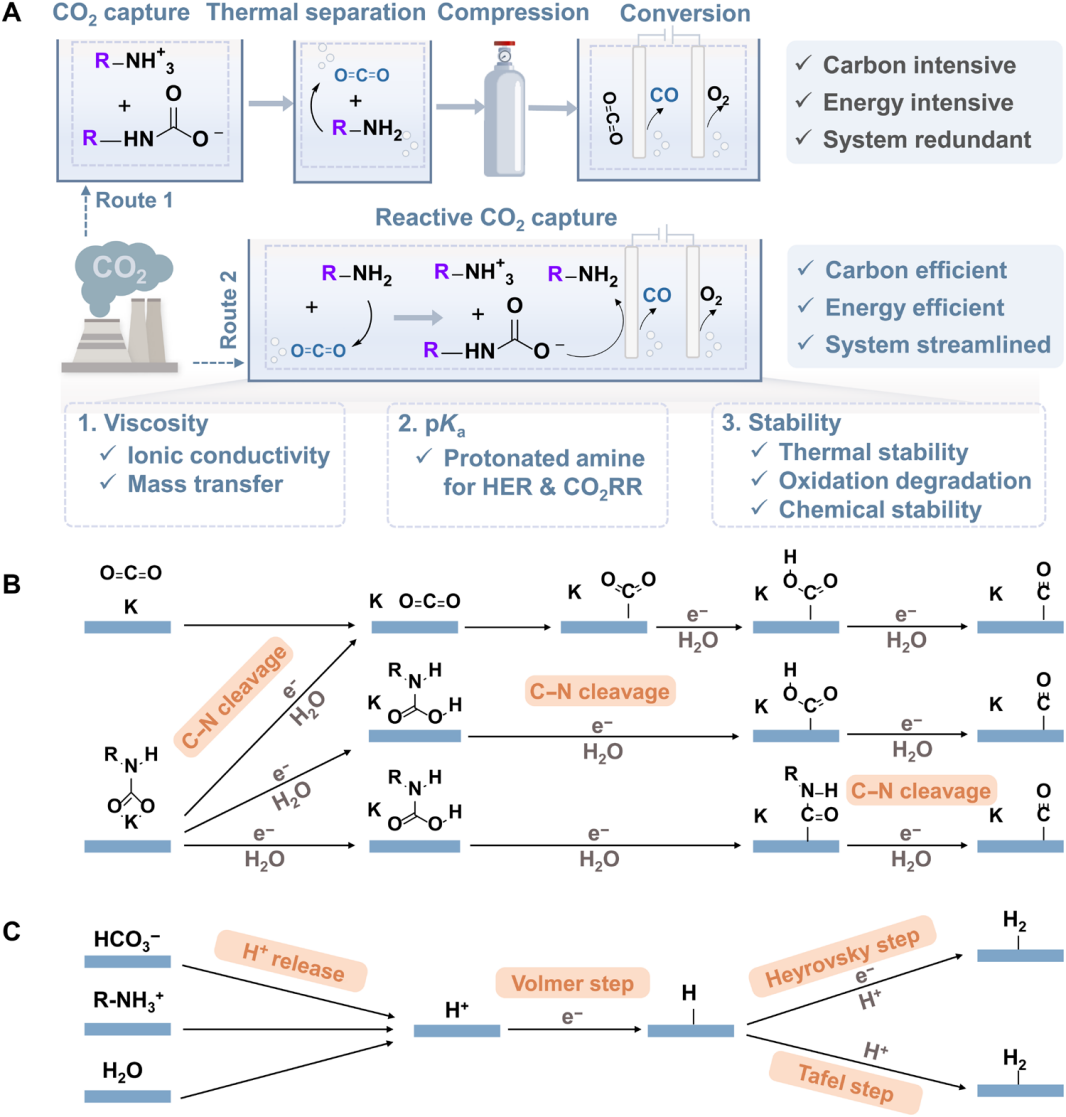
sp^3^-N–mediated reactive CO_2_ capture. (**A**) Conventional gas-phase CO_2_ electrolysis (route 1) versus reactive CO_2_ capture (route 2) and proposed amine screening metrics. Proposed reaction mechanisms of cathodic (**B**) CO_2_RR and (**C**) competitive HER.

Unlike conventional gas-phase CO_2_ reduction, which relies on high energy consumption to extract high-purity CO_2_ gas, reactive capture electrochemically reduces CO_2_ directly from captured solutions. For this integrated electrolysis scheme, sp^3^-N amines such as ethanolamine, AMP, etc., are first used as the capture agents owing to their wide utilization in the conventional amine scrubbing field. Consequently, amines both act as CO_2_ capture agents to covert CO_2_ into liquid carbon species and serve as the electrolyte in the subsequent electrolysis step. This integrated approach bypasses energy-intensive CO_2_ separation and enrichment steps, offering high energy efficiency. In addition, it eliminates redundant systems and complex operational processes, making it highly feasible for industrial applications (Fig. 6A). Given the complicated solution species and electrolysis chemistry in this very pioneering field, several serious issues still challenge the whole community. First, most of the early studies reported that the CO FE was lower than 50% (standard conditions) in the electrolysis of MEA- and/or AMP-captured CO_2_ solutions even by using benchmark Ni- and/or Ag-based catalysts. This implies that the mass transfer dynamics for integrated CO_2_ capture and conversion is probably different from the gas CO_2_ electrolysis counterpart. Meanwhile, the conventional Ni- and Ag-based catalysts might not perfectly match the integrated CO_2_ capture and conversion field (*82*). Second, the HER is rather difficult to suppress in this field compared with CO_2_ gas-phase electrolysis because of the complex and dynamic proton species distributions including protonated amine, bicarbonate, and water. Different from the conventional gas-phase CO_2_ reduction reaction (CO_2_RR) in which HCO_3_^−^/OH^−^ acts as the main proton donors, all these proton species, especially with the addition of tertiary ammonium, would probably be favorable for the HER and/or participate directly in proton-electron transfer to CO_2_ in the CO_2_R, which makes the electrode-electrolyte interface more complex and the initial reaction pathway difficult to distinguish. Third, at its very early development, it is very controversial regarding the role of direct reactants (gaseous CO_2_ or liquid species) and their thermodynamic and kinetic properties. The point that (bi)carbonate cannot be directly reduced but potentially replenish dissolved CO_2_ in the gas-phase or (bi)carbonate electrolysis system has been widely discussed. However, whether carbamate can be either (neither) directly reduced or (nor) just release CO_2_, or both, into electrolytes when the mass transport of dissolved CO_2_ is limited remains unknown. Regarding these key milestones stepping from the lab toward industry for this integrated route, understanding the underlying reaction mechanism and addressing fundamental scientific issues are crucial for optimizing the electrodes, electrolytes, and other system components.

#### Rationale of capture absorbents

Apart from these most important metrics of amines for scrubbing, low volatility, high thermal and chemical stability, and nonpoisonous characteristic are preferable to have excellent overall performance. On the basis of this principle, the optimal capture absorbents for this integrated capture and electrolysis scheme are those having the lowest overall energy consumption (mainly electrolysis energy) and best capture performance. In terms of capture performance, the capture rate and capture capacity can be selected, which are akin to the thermal scrubbing capture section. For the electrolysis aspect, we tentatively propose a range of parameters that might be deserved to do in-depth studies for the research community (Fig. 6A). (i) Viscosity: it measures a fluid’s resistance to flow and the internal friction between molecules. In electrochemical systems, it is crucial to pick up an appropriate amine with excellent viscous properties as it affects the ionic conductivity, mass transfer, and stability of reaction sites. It is challenging for specific amine solutions to have fixed viscosity values as it is highly dependent on molar concentrations, amine mixing ratio, and conditions (temperature and pressure). High viscosity would bring reduced ionic conductivity and limited ion migration, thus reducing the CO_2_ reduction current density. In addition, hindered CO_2_ mass transfer would also occur and thus slow down the diffusion rate of CO_2_ in amine solution, making it harder for the reactant to reach the electrode surface quickly. In contrast, low viscosity implies faster ion diffusion and conductivity for water molecules, amines, and amine-CO_2_ adducts, which might lead to accelerated HER kinetics. Furthermore, in practical experiment manipulations, we found that lower viscosity generally leads to easier stirring and can create turbulence near the electrode surface, causing an uneven reaction environment. (ii) p*K*_a_: The higher the p*K*_a_ value of an amine, the weaker its ability to donate protons. In early efforts, it has been demonstrated that protonated amines with different p*K*_a_ values serve as competitive proton donors and have distinct HER activity (*78*). For instance, they observed that ammonium cations like protonated dimethylethanolamine (DMAEH^+^, p*K*_a_ = 9.2) and β-aminopropionitrile (BAPNH^+^, p*K*_a_ = 7.8) were more favorable proton donors than HCO_3_^−^ (p*K*_a_ = 10.3) for the HER, while protonated MEA (MEAH^+^, p*K*_a_ = 9.5) and AMP (AMPH^+^, p*K*_a_ = 9.7) may be comparable or even worse proton donors. Furthermore, in the following work, the research community recommended using a proton source with low p*K*_a_ and an appropriate metal catalyst to suppress HER (*83*) because ammonium species would impede CO_2_ reduction by covering on the Cu electrode surface, whereas they enhanced the production of hydrocarbon products by regulating proton shuttling on Pb electrodes (*82*). In practical implementation, it seems difficult to give determined p*K*_a_ values when selecting amines because of the dynamic process and complex speciation. As summarized above, we propose that the relationship between the chemical structure and the p*K*_a_ values of ammonium cations, as well as their efficacy as proton donors in suppressing HER and enhancing CO FE, plays a key role. (iii) Stability: Stability is also a fundamental and crucial criterion in the integrated route because thermal stability, oxidative degradation, and chemical stability are required in electrochemical amine regeneration. First, from the thermodynamic aspect, the implementation at higher temperatures can lead to the leaching of evaporative loss of the amine content. In general, amines with strong hydrogen bonding have lower volatility because these bonds increase intermolecular attraction, making it harder for the molecules to escape from the liquid phase. Along with the thermal stability, oxidative degradation is a key property that should be taken care. It has been demonstrated that amines (e.g., polyamines) may undergo oxidative degradation to chain cleavage to produce oxidation byproducts like amide or imide, which would interfere with the interfacial microenvironment and the catalytic performance. Last, the CO_2_-induced chemical stability effect should not be overlooked for the amine-CO_2_ complex because repeated CO_2_ absorption-desorption cycles are required. We recommend three parameters such as a substantial drop in pH, solubility change, and decline in CO_2_ absorption capacity after multiple cycles to consider its chemical stability.

#### Authentic reactant species: Gas molecules versus liquid species

In gas-phase CO_2_RR scenarios, it is widely agreed that dissolved or evolved free gaseous CO_2_ molecules act as the reactant species (*84*, *85*). However, for the integrated amine-capture and electrolysis system, identifying the dominant reactant species is still challenging because the captured carbon species exist in different molecular configurations with dynamic equilibria (carbonate/bicarbonate, carbamate, gas CO_2_ molecules, etc.). In this section, we will focus on the clarifications of key reactant species in this integrated amine-capture and electrolysis system. Because of the sluggish release of CO_2_ from liquid carbamates with strong N─C bonding, the direct electrolysis of amine-CO_2_ solutions is subject to sluggish reaction activity and slow conversion rates. On the basis of the major concerns of the electronegative property of carbamate and low electrolysis efficiency, the main research community considers the free dissolved gas CO_2_ as the reactant. In 2017, Chen *et al.* (*86*) experimentally compared the HER in 30 wt % MEA aqueous solution with CO_2_ loading values of 0.3, 0.4, and 0.48 mol CO_2_/mol MEA, where only different concentrations of carbamate existed, showing an independency of conversion efficiency on CO_2_ loading. They excluded the possibility of direct reduction of carbamate and further concluded that free dissolved CO_2_ was the primary reactant. Different from the MEA that mainly generated carbamate after CO_2_ capture, tertiary amines capture CO_2_ and only form bicarbonate, which would help us better comprehend the reaction pathway. In 2018, Diaz *et al.* (*87*) achieved the direct electrolysis of 1-cyclohexylpiperidine (CHP)–captured CO_2_ solutions where only HCO_3_^−^ species exist. This work clearly demonstrated that the dissolved CO_2_ along with bicarbonate contributed to the carbon source. In 2021, Pérez-Gallent *et al.* (*88*) presented the electrolysis of AMP in propylene carbonate solution without the proton (e.g., water) interference. By comparing the reduction current in N_2_-saturated amine-CO_2_ solutions with CO_2_-free amine solutions, their similar reduction current density profiles indicated that there was barely a reduction reaction. This trend suggested that carbamate/bicarbonate species were not themselves directly reduced because propylene carbonate did not contribute any protic species to HER. In 2022, Ahmad *et al.* (*89*) and Langie *et al.* (*90*) both claimed that bicarbonate acted as the carbon source to liberate the CO_2_ to the cathode vicinity before electrochemical reduction in 1 M AMP and 3 M triethylamine (TREA) aqueous solutions, respectively. To address discrepancies regarding amine types and their roles, in 2023, Leverick *et al.* (*91*) unambiguously indicated that dissolved free CO_2_, rather than carbamate, was the active species by manipulating CO_2_ partial pressure, p*K*_a_ values, and amine structure. In short, on the basis of the current progress, it is becoming clearer that the dissolved free CO_2_ acts as the direct reactant, while bicarbonate/carbamate contributes to the carbon source as CO_2_ is consumed upon reduction. However, another perspective claims that direct C─N bond cleavage in carbamate is possible and even suggests that carbamate itself acts as the reactant. In 2019, Khurram *et al.* (*92*) mixed 2-ethoxyethyl-amine (EEA) with the organic solvent dimethyl sulfoxide (DMSO) to avoid the side effect of the HER in aqueous solutions and further introduced varied cations, aiming for the direct electrolysis of carbamate salts. They found that the N─C bond length increased from 1.38 to 1.40 Å and O─C─O bond angles reduced from 125.1° versus 122.3°, following the order from Li^+^ to K^+^-carbamate salts. This change in bond length and bond angle implied that alkali carbamates became easier to be directly reduced than neutral carbamic acid. In addition, alkaline cations were capable of ion transfer coupling with charge transfer to realize the direct electrolysis of amine-CO_2_ adducts because electron transfer alone to carbamate cannot lead to N─C bond cleavage. In addition, the low efficiency of carbamate electrolysis may be attributed to sluggish electron transfer from the electrode to carbamate, which inspired the research community to improve reactant adsorption energy by enhancing the electric field strength. Inspired by this, in 2021, via systematic tuning of the electrochemical double layer (EDL) structure, Lee *et al.* (*93*) achieved much higher CO FE by introducing competitive adsorptive K^+^ with the MEAH^+^ ions for surface binding sites to improve the electron transfer to carbamate in MEA/KCl electrolytes. The introduction of K^+^ led to a compacter EDL and thus strengthened the local electric field. In 2023, Shen *et al.* (*94*) combined experiments and theoretical calculations to demonstrate that dissolved free CO_2_ was before direct reduction, while carbamate reduction became feasible at notably negative potentials. To support the prospective, the calculated reaction free energy of CO_2_(g) absorption at 1.3 V versus SHE was +0.36 eV, while those of the C─N and C─O bond cleavage of carbamate were +0.45 and 0.49 eV, respectively. In 2024, Neves-Garcia *et al.* (*95*) achieved the direct electrochemical conversion of carbamate to CH_4_, providing evidence that carbamate, rather than (bi)carbonate or dissolved CO_2_, served as the primary reactant. At the early development of the integrated amine-based system, the argument about the reactant and the underlying mechanism is ongoing and the countless amines that generate kinds of carbamates also bring about much more complexity to this classification. In addition, the good match between carbon species (liquid carbon species versus gas-phase CO_2_ molecules) and the catalytic microenvironment (e.g., metal catalysts and supporting electrolytes) is likely to contribute to the identification of reactive reactants.

#### Possible reaction mechanisms

CO_2_ has a linear and centrosymmetric structure, with C═O bonds broken by high-energy electrons and protons during electrolysis in aqueous solutions. In the electrochemical reduction of amine-captured CO_2_, protons and carbon sources compete at the cathode, while the OER occurs at the anode. The carbon source reduction pathway is proposed as two reduction mechanisms (Fig. 6B). The first reduction pathway for dissolved CO_2_ follows the conventional CO_2_ electrolysis mechanism, including CO_2_ adsorption, surface diffusion, two proton-coupled electron transfer (PCET) steps, and product desorption. Throughout this process, the C═O bonding is strongly perturbed by the substrate, sharing electrons with the catalyst. Neutral hydrated CO_2_ molecules are converted into CO_2_*^−^, which reacts with H_2_O molecules to form HCO_2_*. Because of its instability, HCO_2_* is reduced to HCO_2_^−^, which desorbs from the catalyst. In the second pathway of carbamate reduction, carbamate can either decompose via C─N bond cleavage with a proton-electron transfer, generating CO_2_ for typical CO_2_ reduction, or undergo direct proton-electron transfer to form the RNHCOOH* intermediate, preserving the C─N bond. In the first case, the newly formed CO_2_ is reduced as in the conventional CO_2_RR. In the second case, the RNHCOOH* intermediate can either break the C─N bond to generate COOH* or undergo further reduction of RNHCO* through proton-electron transfer. Thus, C─N bond cleavage and preservation coexist in carbamate reduction. Because of the lack of an appropriate charge transfer pathway, reduction requires the aid of cations in the double layer to strengthen the local electric field and facilitate electron transfer. In reference to the competitive HER pathway, the internal mechanism remains a debate because of competing proton sources and synergistic pathways (*96*). We propose that the p*K*_a_ values of the proton source likely determine the effective proton donor including H_2_O, HCO_3_^−^, and RNH_3_^+^, which are highly related to the HER process. During HER, a proton from these sources adsorbs on the catalyst surface to form an adsorbed H* (Volmer step) (*97*), and the adsorbed H* combines with a H^+^ and an electron (e^−^) to form a H_2_ molecule (Heyrovsky step or electrochemical desorption step) (*98*). Alternatively, H_2_ molecules can be formed through the Tafel step, where two H* combine on the catalyst surface (Fig. 6C).

#### Strategies for enhancing reactive CO_2_ capture

In its infancy, the research community primarily focused on system electrolysis efficiency in terms of electrode selection, electrolyte design, and electrolyzer engineering and has accumulated substantial experience. In this section, we highlight effective approaches from these three perspectives, aiming to pave the way for advancing reactive CO_2_ capture.

### Electrode

**Fig. 7.**
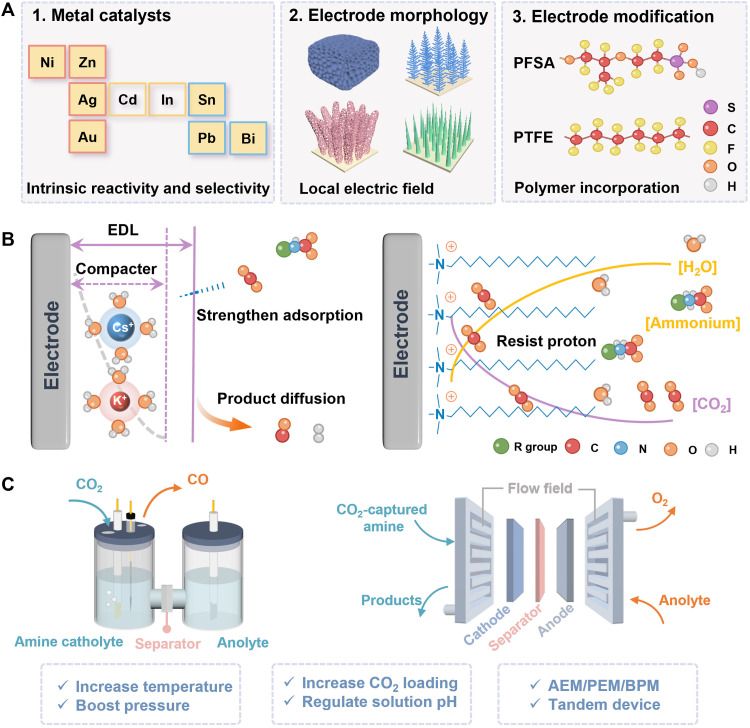
Strategies for promoting sp^3^-N–mediated reactive CO_2_ capture. (**A**) Electrode engineering from the aspects of metal selection, morphology control, and additive functionalization. (**B**) Electrolyte engineering via incorporation of metal ions and molecules. (**C**) Electrolyzer engineering that focuses on the optimization of components and working conditions.

Electrocatalysts play a decisive role in stabilizing the reaction intermediate, reducing the reaction barrier, and optimizing reaction pathways. On the basis of Sabatier’s principle (*99*), the catalyst-intermediate interaction strength is highly dependent on the geometrical structures and electronic configurations of the catalysts, which in turn affect the catalytic reactions positively or negatively. Therefore, many classical studies focused on the employment of various metal entities from noble metals to transition metals because of their different d/p orbital electronic structures (e.g., Au, Ag, Ni, Cu, Bi, etc.) and varied catalyst-intermediate interactions (Fig. 7A). For the first strategy, in the multistep electron-proton transfer process, the catalysts with different binding strengths for the reactant, key intermediate species, or products are responsible for the desired product selectivity (e.g., C_1_ and C_2+_) and desorption rates, whereas the binding energy with intermediates is highly dependent on tailoring the choice of electrode materials. For instance, Bruggeman *et al.* (*82*) investigated the reaction pathway mediated by the carbamate bond strength, proton shuttling, and amine structure (MEA versus AMP) on Cu (inner-sphere electron transfer) and Pb (outer-sphere electron transfer) electrodes. They indicated that Pb electrodes were more suitable for direct carbamate reduction, whereas substrate adsorption on the surface was not beneficial. Alternatively, one of the major phenomena that emerge in amine-based electrolysis is the HER prevalence, which is correlated to the key species such as protonated amine and captured CO_2_. To investigate the direct correlation between binding energies and CO_2_R intermediates, an in-depth investigation of activity and stability has proceeded using Cu, Ag, Au, Sn, and Ti electrodes, combining experiments and calculations (*100*). The result showed that (i) HER dominated in Cu and Sn metals, (ii) Au and Ag preferred activity for dissolved free CO_2_, and (iii) Ti only generated hydrogen. This work provided fresh insights into the codesign of binding energy between capture agents and heterogeneous catalysts in integrated systems. Different from the discussed bulk metal catalysts, the molecule/atom catalysts [e.g., single-atom catalyst (SAC)] have distinct active centers and energy barriers with intermediates because of variable coordination environments like bonding length, high/low coordinated atoms (M─N*_x_*), etc. (*101*). This inspired the following work to expect the capabilities of atomic catalysts in amine-based scenarios. Thus, specific cation-insensitive Ni─N─C SAC was prepared and achieved a high CO selectivity of 64.9% at −50 mA cm^−2^ in CO_2_-saturated MEA-H_2_O electrolytes via membrane assembly electrode reactors (*102*). This Ni SAC showed superior intrinsic activity toward stabilizing the CO_2_^−^ intermediate regardless of the bulkiness of cation types and the high energy barrier for the HER. Furthermore, the surface charge density on the cathode was influenced by the bulkiness of cations, which varied between amine structures, sizes, functional groups, etc. For the second strategy, electrode morphology control offers another way to regulate the catalyst’s performance by changing the porosity, local electric field, and beyond. Chen *et al.* (*86*) designed electrodes with porous and coralline-like structures to enhance mass-transport kinetics. Their design resulted in a formate FE of 60.8% on the porous Pb electrode, which was notably better than 2.4% on the coralline-like electrode. The first work provided the catalyst design of electrode morphology engineering for improving electrolysis efficiency and product distributions in the amine system. Afterward, a three-dimensional coral-Ag/C structural catalyst with excellent hydrophilic characteristics was synthesized (*90*). They concluded that the three-dimensional coal carbon support with randomly distributed cores improved the electrolysis performance by enhancing the surface binding affinities for CO production, which resulted from sufficient interactions at the catalyst-CO_2_ interfaces. Hossain *et al.* (*103*) reported that in their work, nanodendrite electrodes were prepared by directly growing coinage metal (Cu, Ag, and Au) nanodendrites on glassy-carbon substrates. These electrodes showed higher current densities, lower onset potentials, and reduced charge-transfer resistances when compared to smooth structures in CO_2_-saturated MEA electrolytes. Furthermore, they found that morphological electrodes induced a localized high electric field and in turn caused a high local concentration of CO_2_, consequently causing a reaction overpotential decline and reaction rate improvement. Last but not least, incorporation of a range of molecules with hydrophobic groups such as polytetrafluoroethylene (PTFE) and perfluorinated sulfonicacid (PFSA) ionomers to manipulate the gas and water transport provides more possibilities for tuning the selectivity toward certain products. It is known that gas transport is promoted by hydrophobic molecules, while water or ion transport is dominated by hydrated hydrophilic domains. PTFE has been widely used in the gas-phase CO_2_RR to tailor the electrode-electrolyte interface for an optimal reaction environment, which also provides us a possible way in integrated electrolysis. The research community reported that they designed a reticulated catalyst with a composition of Ag/Nafion/PTFE (8/1/1) for the direct electrolysis of CO_2_-saturated aqueous 1.25 M CHP solutions (*87*). The result showed that a CO FE of 71.7% at current densities of 104 mA cm^−2^ was achieved with the addition of 0.2 M K_2_SO_4_ additives under a back pressure of 20 psig (pounds per square inch gauge). This improvement can be attributed to the suppressed proton adsorption and enhanced mass transport, which were induced by the hydrophobic local environment with the addition of PTFE. Another impressive model is PFSA, which combines hydrophobic and hydrophilic functionalities along with excellent ion transport. Sargent and colleagues (*104*, *105*) developed a catalyst:ionomer (PFSA) bulk heterojunction architecture to enhance the gas diffusion in gas-phase CO_2_ electrolysis. The works demonstrated that the hydrophobic and highly charged hydrophilic domains on PFSA diminished hydrogen adsorption and enhanced CO_2_ availability, thereby increasing the current density. In the future, the macromolecule PFSA would probably be applicable in an amine-based system regarding the HER, limited local reactant, and low current density. In all, electrodes modified by hydrophobic species may be helpful to improve the local concentration of reactants, hinder the proton source, and thus suppress competitive HER.

### Electrolyte

The electrode reaction kinetics is highly affected by the electrode-electrolyte interface structure, which can be precisely modulated through the changes in the active species types, concentrations, structures, etc. Figure 7B presents the efforts the research community has made to regulate the electrode-electrolyte interface structure and electrolyte chemistry by adding alkali metal ions (e.g., Cs^+^, K^+^, Na^+^, and Li^+^), charge-neutral additives [e.g., cetyltrimethylammonium bromide (CTAB)], and so forth. Specifically for alkali metal ions, it is widely accepted that their structural solvation can stabilize the CO_2_^−^ intermediate via short-range electrostatic interaction in a local electric field and increase the local concentration of dissolved CO_2_ by buffering pH near the cathode. In amine solutions, the steric adsorbates (e.g., size, length, and types of molecular chains) at the electrode interface influence the orientation and conformation of the EDL and electron transfer dynamics, which are ascribed to charge transfer across ammonium cations before reaching carbamate. On this basis, the roles of K^+^ in a 30 wt % aqueous MEA-CO_2_ electrolyte (saturated with N_2_) on the tailoring thickness of EDL and subsequent electrochemical activity were investigated (*93*). The result indicated that the K^+^ ion with a larger cationic size competed with the MEAH^+^ ion for surface binding sites to construct compacter EDL, resulting in the enhancement of the strength of the interfacial electric field and the improvement of the adsorption energy of the reactant. Moreover, the thicknesses of EDL for K^+^, Rb^+^, and Cs^+^ were lower than those for MEAH^+^, Li^+^, and Na^+^ because of their smaller cationic sizes. As discussed before, carbamate electrolysis is difficult because of the strong N─C bond with a very short N─C length. After indicating the amine-activation process in the former work (*106*), the cation-solvent-amine interactions and conversion kinetics were reported by incorporating alkaline cations (K^+^, Na^+^, and Li^+^) into EEA in DMSO solvents (*92*). The EEA/DMSO/K^+^ electrolytes showed the largest reduction currents and reaction rates, which may result from the faster K^+^ transfer from the bulk solution to reaction sites. For another aspect of charge-neutral additives of CTAB, earlier works have demonstrated its hydrophobic function in tailoring gas availability and mediating the water microenvironment as electrolyte additives in MEA solutions (*86*). For a further study, Ahmad *et al.* (*78*) introduced CTAB into aqueous 1 M AMP solutions and achieved enhanced product selectivity up to 1.7 times that in a 1 M MEA electrolyte. This improvement was attributed to more repulsion for AMPH^+^ and water molecules to suppress HER and a lower energy barrier for nucleation because of the incorporation of CTAB. It was revealed that the hydrophobic-aerophilic interface microenvironment triggered by CTAB regulates the interfacial water environment by repelling proton sources and suppressing the water orientation as well as promotes CO_2_ enrichment at the electrified interface, increasing the selectivity of CO_2_ electroreduction to CO. Beyond that, other additives like highly electronegative Cl^−^ ions have been demonstrated to be effective in facilitating the transport of more CO_2_ to the catalysts and led to better CO_2_RR performance, which could be probably promising in amine-based electrolysis. From the above, the introduction of alkaline cations or surfactants into amine electrolytes is beneficial to improve local electric field intensity and stabilize the chemical microenvironment for charge transfer and mass transport kinetics.

### Electrolyzer

H-type cells and membrane-electrode assembly reactors are two important devices also in the field of integrated CO_2_ capture and conversion for evaluating broad electrochemical performances (*107*). An H-type cell with a three-electrode configuration is normally used to evaluate fundamental electrochemical parameters and extract reliable reaction kinetics data under low current density conditions. It consists of a cathode chamber and an anode chamber separated by an ion-exchange membrane (Fig. 7C). However, the H-cell suffers from mass transfer limitations and large solution resistance, yielding high overpotential and low energy efficiency. Meanwhile, for MEA electrolyzers, they are usually composed of a separator, catalyst layers, a gas-diffusion layer, flow channels, and bipolar plates (Fig. 7C). This zero-gap configuration allows the easy scale-up tests at large current densities, low cell resistance, and voltage and hence opts to be a rather practical solution working under industrial-demanded conditions. Therefore, in terms of amine solution electrolysis, an H-cell is beneficial to concentrating on the fundamental research of the cathode reaction, while a MEA cell has great potential for the following scalability and potential for the scale-up (*108*). We next discuss how to possibly further improve the overall integrated CO_2_ capture and electrolysis performance from the aspect of a MEA cell electrolyzer. The first should be the working condition optimization including internal electrolysis parameters and external system operating conditions. The operation of the MEA cell starts feeding CO_2_-rich amine solution into the cathode inlet and pumping reduced products and CO_2_-lean amine solution to the container. The amine-CO_2_ interaction is highly dependent on external operational factors such as pressure, temperature, and local pH. Specifically, increasing the solution temperature and operating pressure can facilitate the breaking of the N─C bond and accelerate gaseous CO_2_ release to the interfacial electrode for a high local CO_2_ concentration. Lee *et al.* (*93*) heated the 3 M MEA-CO_2_ aqueous catholyte from 40° to 80°C and achieved 15 times higher current density at 60°C than that at room temperature. Furthermore, Kim *et al.* (*102*) also investigated the CO_2_ regeneration rate as well as the CO_2_ RR and HER kinetics in MEA solutions by increasing the reaction temperature from 5° to 60°C. They found that the activation of carbamate for CO_2_ conversion was not enough at low temperature, while the CO partial density showed the maximum at 40°C and then decreased at 60°C. This volcano trend may be attributed to HER being rapidly generated at high temperatures, implying that the electrolysis performance in amine-based solutions is the trade-off activation of carbamate and competitive HER. On the internal hand, according to Henry’s Law (*109*), the strategy of boosting the CO_2_ partial pressure to increase the CO_2_ loading capacity can be used to strengthen CO_2_ solubility in amine-based solutions. In this process, the introduction of acid CO_2_ into basic amine-based solutions always swings solution pH values and brings about a dynamic chemical microenvironment. On the basis of these perspectives, Leverick *et al.* (*91*) introduced different CO_2_ concentrations—800 ppm, 1 vol % CO_2_, 10 vol % CO_2_, or 100 vol % CO_2_—into 2 M MEA for electrolysis. They observed that the pH exhibited a decreasing trend, while CO FE showed a first-order dependence on CO_2_ partial pressure (CO_2_ loading). This investigation unveiled that feeding diluted/concentrated CO_2_ would increase CO_2_ loading, lower the local pH, and thus increase electrolysis performance. In practical manipulation, optimizing the external and internal operation conditions would be much effective and should be precisely controlled in terms of amine types, catalysts, devices, etc.

The second aspect is the wise selection of separator or optimal system engineering. The employment of a separator in the MEA electrolyzer aims to conduct ions for fast reactions. The most common separators in the MEA configuration are the proton exchange membrane (PEM), anion exchange membrane (AEM), and bipolar membrane (BPM), featuring conducting cations (e.g., H^+^ and K^+^) with high conductivity and mobility and anions (e.g., OH^−^ and HCO_3_^−^) as well as enabling simultaneous H^+^ and OH^−^ production to the cathode and anode through water splitting, respectively. Thus, from the architecture aspect, BPM can be considered as a combination of AEM and PEM at the interfacial layer, which can inhibit ion flow between the cathode and the anode. Hence, the choice of suitable separator according to the solution chemistry to achieve maximized overall electrolysis energy efficiency deserves to be better studied. In practical implementation of electrochemical reduction of amine-captured CO_2_, AEM is commonly used because it can transport OH^−^ ions for local basicity to promote the CO_2_RR, prevent H^+^ ion migration to the cathode to suppress HER, and reduce catalyst reconstruction (e.g., Ni, Zn, Ag, etc.). However, this configuration often leads to notable liquid carbon crossover known as the “carbonate problem” and “carbamate problem” and delivers lower current densities and FE when compared to gas-phase electrolysis. Earlier work has demonstrated that the protonation of (bi)carbonate at the cathode to release CO_2_ using BPM was feasible and 100% carbon utilization in electrochemical reduction of carbonate solutions was achieved (*110*). In this regard, Langie *et al.* (*90*) manipulated the BPM separator in a 3 M TREA electrolyte [only (bi)carbonate formation] and achieved a CO FE of 35% at −100 mA cm^−2^, maintaining stable operation for 70 hours, much higher than that in AEM or PEM. They found that high H^+^ flux from self-dissociation of water catalyzed by BPM could be delivered to the cathode side to trigger a reaction with (bi)carbonate for more CO_2_ release. We would propose that BPM may be helpful for the improvement of amine electrolysis where only bicarbonate generates as bicarbonate is more favorable for direct conversion of CO_2_ compared with carbamate. However, BPM always brings out drawbacks such as high overpotential and low energy efficiency because of the required applied voltage of >0.83 V for water splitting, which limits the potential industrial environment (*111*).

The last one is the emerging multicomponent paradigm known as the tandem electrocatalytic reactor that decouples individual steps within a chemically complicated pathway via multicomponent design. The current tandem device scheme generally consists of two consecutive cells: CO_2_ is reduced to CO in the former reactor, and CO is transferred into the second reactor to undergo subsequent C─C bond formation for valuable products such as acetate, C_2_H_4_, or other C_2+_ products (*112*). This CO_2_RR-COR scheme promotes electrocatalytic activity and targets product selectivity as it can precisely control two separated reactions at different pH values (*113*). For instance, Sargent and colleagues (*114*) reported decoupling the CO_2_-to-C_2+_ reaction into spatial distinct two steps, CO_2_-to-CO and CO-to-C_2+_, by coupling Co-based and Cu-based catalysts in a tandem device and achieved a single-pass carbon efficiency of 90 ± 3%, simultaneous with a C_2_H_4_ FE of 55 ± 3% and a total C_2+_ FE of 76 ± 2% at 800 mA cm^−2^. Beyond tandem electrolysis, the tandem processes could also couple a mature thermocatalytic or biocatalytic reaction with an electrocatalytic process under different operating temperatures, pressures, and pH values. To the best our knowledge, there are less reported works about coupling amine-captured tandem electrolysis as the liquid carbamates are generally not electrochemically active along with the much low CO selectivity. However, we consider it a burgeoning technique that is highly promising for the CO_2_ conversion community and worth highlighting here. Future research efforts could focus on enhancing CO_2_-CO selectivity and promoting CO electrolysis toward a single product, aiming to overcome the challenges of low multicarbon selectivity and low single-pass carbon efficiency in C_2+_ product conversion.

## SP^2^/SP^3^-N–MEDIATED GAS-PHASE CO_2_ CONVERSION

Gas-phase CO_2_ electrochemical conversion is a relatively mature technology that converts gaseous CO_2_ into valuable products in aqueous systems with the input of electrical energy and pure CO_2_ gases. The past decades witnessed the great advancement of gas-phase CO_2_ electrolysis in terms of reduced products, product selectivity, working current density, etc. (*115*, *116*). However, it still faces the grand challenges of being mainly limited to C_1_ products, competitive HER, carbon crossover, and sluggish CO_2_ adsorption and reaction kinetics. A lot of classical regulation strategies that focused on catalysts (e.g., alloying and doping) (*117*–*119*), triple-boundary interfaces (e.g., microenvironment engineering) (*120*), and reaction conditions (e.g., reactors, membranes, temperature, and pressure) has been developed, and great achievements have been obtained (*121*). However, these regulatory strategies introduce excessive complexity, limited scalability, intricate reaction mechanisms, and higher system costs. Given their strong CO_2_ affinity and tunable electronic structures, sp^2^/sp^3^-N structures are promising catalog materials that will play an increasingly important role in gas-phase CO_2_ electrolysis.

### Promoting adsorption and mass transfer

**Fig. 8.**
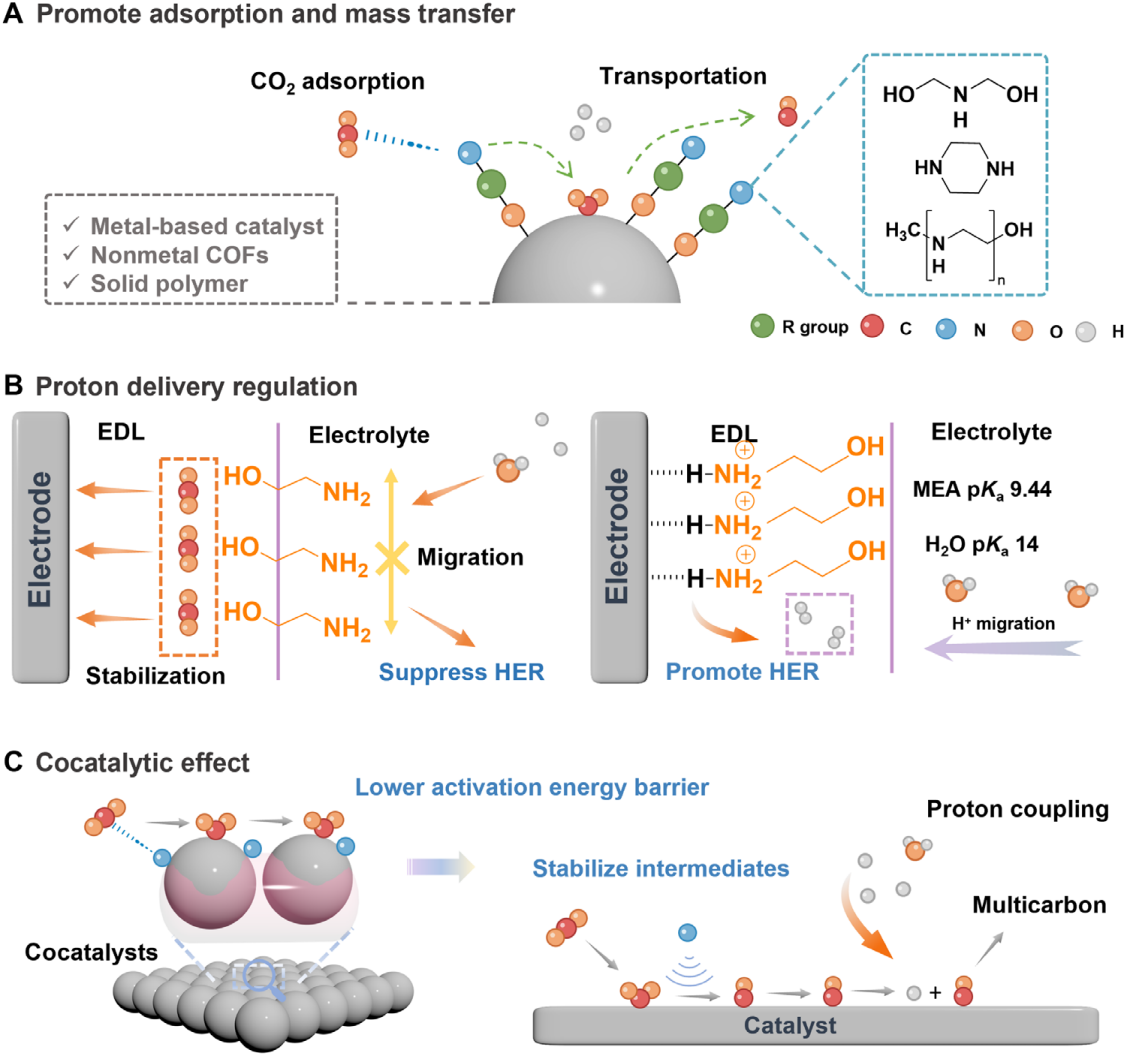
Function of sp^2^/sp^3^-N structures in the mediation of gas-phase CO_2_ conversion. (**A**) Promoting CO_2_ gas adsorption and mass transfer. (**B**) Proton delivery regulation by incorporating sp^2^/sp^3^-N compound additives into electrolytes. (**C**) Cocatalytic effects.

CO_2_ gases have a problem of low solubility in aqueous electrolytes (e.g., 0.03 M in 0.01 M KHCO_3_), leading to a serious issue of insufficient local CO_2_ reaction concentration (*122*). Moreover, its stable molecular structure confers CO_2_ gases with low reactivity, which are hardly activated at the catalyst’s surface. To this end, creating a microenvironment that increases the local CO_2_ concentration, promotes CO_2_ activation, and/or strengthens CO_2_ adsorption and mass transfer steps is of vital importance for further improving the gas CO_2_ electrolysis overall performance. Building a delicate triple phase boundary by incorporating additional hydrophobic polymers (e.g., PTFE) is a good strategy for enhancing local CO_2_ concentrations, which has been widely used in the past years (*123*, *124*). sp^2^/sp^3^-N structures have a stronger interaction tendency with CO_2_ molecules, and the incorporation of them might also contribute to accelerated CO_2_ adsorption as well as mass transfer steps. Meanwhile, the chemical interaction between amine and CO_2_ could theoretically bring faster CO_2_ mass transfer steps than a physical trapping manner by using PTFE, and this should result in better overall electrolysis performance. Furthermore, the surface functionalization approach to tuning the catalytic microenvironment has been demonstrated to be feasible for the CO_2_RR (*125*). On the basis of this concept, the research community attempted to functionalize catalysts using sp^2^/sp^3^-N compounds to strengthen CO_2_ adsorption and promote mass transfer (Fig. 8A). Pioneering work in 2016 demonstrated that amino acid grafting on Cu electrodes could double FE in CO_2_ reduction to hydrocarbons, with HER being entirely suppressed (*126*). This work informed researchers that sp^3^-N compounds could be promising modifiers for improving CO_2_ electrolysis activity, a strategy that deserved to be extended to other types of sp^2^/sp^3^-N molecules. In light of these findings, diethanolamine (DEA) was grafted on metal-based tin oxide to obtain amine-functionalized tin oxide catalysts (DEA-SnO*_x_*/C) for the direct electrolysis of dilute CO_2_ (*127*). This catalyst achieved a maximum formate FE of 84.2% at −0.75 V (versus reversible hydrogen electrode) with a partial current density of 6.7 mA·cm^−2^ in 0.5 M KHCO_3_ under simulated flue gas conditions. The capability of CO_2_ electroreduction to operate at low CO_2_ concentration was attributed to the enhancement of CO_2_ adsorption, activation, and hydrophilic properties on electrode surfaces functionalized by amino groups. In addition, the amine molecules selectively permeated CO_2_ while blocking O_2_ transportation owing to the strong interaction between CO_2_ and amino groups, which also exhibited good performance for inhibiting O_2_ reduction. This work demonstrated the high feasibility of using amine molecules as surface regulators to manipulate the interaction between catalysts and reactants for the CO_2_RR. These early studies successfully validated the effectiveness of the sp^2^/sp^3^-N compound functionalization strategy, which, on the other hand, inspired the community to hypothesize whether this concept was generally applicable to other nonmetal catalyst systems. Building on prior research, a high-density sp^3^-N catalytic array was developed by embedding PZ into crystalline and microporous amine-linked covalent organic frameworks (COFs), achieving a C_2_H_4_ FE of 19.1%, a substantial advancement in the field (*128*). The negatively charged sp^3^-N sites serving as active centers could accumulate more charges locally to facilitate CO_2_ adsorption and improve the binding capacity for carbon-containing intermediates such as *COOH and *CO. In addition, the sp^3^-N atoms in PZ with strong electron donor properties were conducive to forming hydrogen bonds, stabilizing active intermediates, and promoting C─C coupling (*129*). Besides the above-mentioned catalyst modification, integration of sp^2^/sp^3^-N groups into the membrane would probably be effective in concentrating CO_2_ and enhancing mass transfer. Inspired by this, Aeshala *et al.* (*130*) first synthesized amine-based solid polymer electrolytes by directly blending branched polyethylenimine with poly(vinyl alcohol) and KOH to achieve better CO_2_ electrolysis. The underlying mechanism suggested that CO_2_ molecules were strongly adsorbed by the amino groups at the interface. Apart from the aforementioned works functionalized with sp^3^-N compounds, sp^2^-N structures also show an affinity for CO_2_ molecules and might be used to enhance CO_2_ conversion. Imidazolium is a planar structure of the imidazole ring with delocalized π-electrons over the ring, where the sp^2^-hybridized carbon and nitrogen configuration can be observed. To demonstrate its function for CO_2_ reduction, imidazolium-functionalized polymer electrolytes were developed for gaseous CO_2_ electrochemical reduction (*131*). This work demonstrated the effectiveness of sp^2^-N structures in regulating ion transport and the reaction environment. Inspired by this, Wang *et al.* proposed to functionalize the AEM by mixing poly[(3-methyl-1-vinylimidazoliummethylsulfate)-*co*-(1-vinylpyrrolidone)] (PQ44) with poly(vinyl alcohol) for CO_2_ electrolysis (*132*). They found that the sp^2^-N–hybridized imidazole groups increased the electron cloud density on adjacent carbon atoms owing to the high electronegativity. This enhancement led to a heterocyclic ring with added reactivity, thereby improving CO_2_ adsorption. In all, sp^2^/sp^3^-N structures featuring electron-donating properties can be incorporated into a broad range of catalysts (e.g., metal-based and non–metal-based) to promote CO_2_ adsorption and mass transfer processes. In the future, the molecular structure and anchoring sites of amines are deemed to have multiple effects on the CO_2_RR, as studies have shown that linear amines generally enhance the CO_2_RR, whereas branched amines tend to hinder reactivity (*133*). The effects of substituent groups including electron-donating alkyl groups and electron-withdrawing hydroxyl groups on the CO_2_ capture, the activation of reactant intermediates, and the suppression of the HER deserve to be further investigated.

### Proton delivery regulation

Apart from the concerns of low CO_2_ concentration and limited mass transfer, the parasitic HER in gas-phase CO_2_ electrolysis is another major issue that needs to be carefully managed. The gas-phase CO_2_ electrochemical reduction is essentially a proton-coupled electron transfer process with a CO-producing equation: CO_2_ + H_2_O + 2e^−^ → CO + 2OH^−^. The involvement of protons in the reduction reaction leads to two classical problems: (i) the origination of proton sources and (ii) the competitive water reduction reaction for producing H_2_. The contribution of the proton source from water versus bicarbonate has been a long-lasting argument that can be seen from a lot of articles (*84*, *134*, *135*), which is not our focus here. For another, the research community has been dedicated to suppressing HER by weakening the proton donation capability. Specifically, the common strategies of increasing electrolyte pH (e.g., KOH and NaOH) by introducing buffering solutions (e.g., phosphate and HCO_3_^−^), organic electrolytes, and/or ionic liquids were used to limit the availability of protons and weaken the driving force for HER. However, several drawbacks such as low CO_2_ solubility, decreased conductivity, carbon loss, catalyst instability, and high operating cost would be brought out at the same time. Compared to these common strategies, the incorporation of sp^2^/sp^3^-N molecules into electrolytes to regulate solution species is also interesting (Fig. 8B). To validate this concept, Qiu *et al.* (*136*) used a 0.5 M NaHCO_3_ solution added with 3 mM methyl carbamate (MC) as the electrolyte and first achieved the suppression of the HER as well as a highly enhanced FE for CH_4_ production of 81.6%. For this improvement, they claimed that the addition of sp^3^-N had two functions. First, MC benefited the adsorption of CO_2_ on the active sites and blocked proton interactions, allowing CO_2_ reduction to proceed while weakening competitive HER. Second, sp^3^-N alternatively enhanced the adsorption and stabilization of carbon-containing intermediates. Amines acting as proton scavengers could bind protons and reduce their availability. This was useful in elaborately controlling the local proton transfer at the catalyst-electrolyte interface, thereby suppressing HER and enhancing the CO_2_ reduction process. However, HER would be promoted once the MC concentration was increased to more than 5 mM. Meanwhile, experiment results showed that the primary reduction product in an aqueous electrolyte (0.1 M DEA and 0.5 M KHCO_3_) was H_2_, suggesting that DEA acted as a promoter for H_2_ formation and an inhibitor for CO generation. To further clarify the functions of amine molecules in the proton transport process and the reaction mechanism in the mass-transfer boundary layer, Safipour *et al.* (*137*) conducted gas-phase CO_2_ electrolysis tests by using 1 M KHCO_3_ added with 0.2 M MEA as the electrolyte and a Ag film as the working electrode, respectively. Compared to the blank counterpart (1 M KHCO_3_ without MEA), enhanced H_2_ partial current densities can be observed. The lower p*K*_a_ value of MEAH^+^ (9.44) in comparison to that of HCO_3_^−^ (10.3) implies the stronger proton donation capability, which could be a dominant reason for the highly enhanced HER reaction kinetics in CO_2_ electrolysis. Furthermore, they also speculated that MEA-related species highly likely occupied the EDL and hence was adsorbed to the electrode interface. Consequently, the CO_2_ concentration was largely limited, and the CO_2_-to-carbon performance was decreased. Similar conclusions agreed with the following work (*91*). On the basis of the above analysis, it is reasonable to conclude that amine acts as either a promoter or inhibitor for hydrogen generation during CO_2_ electrolysis, which is highly dependent on the amine concentration, amine p*K*_a_ values, amine molecular structures, etc. (*133*). However, comprehensive research on the potential determinants of proton regulation and the underlying mechanism seems to be lacking. In the future, screening optimal amine molecules for proton management to suppress HER and improve conversion efficiency would be an intriguing area of study for the broad CO_2_ conversion field.

### Cocatalytic effect

In addition to the former two functions, the incorporation of sp^2^/sp^3^-N structures also contributes to cocatalytic effects in gas CO_2_ electrolysis. As a step toward this goal, a hybrid Ag and pyridine cocatalyst was synthesized by electrografting pyridine molecules onto a Ag electrode (*138*). In the following tests, the Ag/pyridine hybrids were found with lower onset potential (200 mV) and 10-fold higher partial current densities compared with bare Ag electrodes. Meanwhile, the pyridine can coordinate and stabilize carboxyl (*COOH), thereby lowering the activation energy barrier of the initial electron transfer (rate-determining step). In addition, the charge delocalization around the nitrogen group of the pyridines promoted the subsequent charge transfer processes. All these cocatalytic factors contributed to the enhanced CO_2_-to-CO performance (Fig. 8C). This work informed the community that product selectivity could be improved by tuning the binding strength of certain intermediates toward high-value products. On the basis of this assumption, Sargent and colleagues (*139*) functionalized a Cu electrode with *N*-aryl-substituted tetrahydro-bipyridine films and achieved an ethylene FE of 72% at −0.83 V, much higher than that of bare Cu electrode (<40%). The test results showed that the sp^2^-N atom of the pyridine ring enhanced the binding strength of *CO, stabilized a higher proportion of *CO on the Cu surface, and lowered the energy barrier of CO dimerization, thereby promoting the CO_2_-to-ethylene conversion. This work demonstrated the cocatalytic function of sp^2^-N compounds in CO_2_ electrolysis by tuning the binding capabilities toward desired reaction intermediates. Drawing inspiration from this concept, researchers hypothesized whether amine molecules have similar cocatalytic behaviors in CO_2_ electrolysis. Bifunctional catalysts functionalized with amines could potentially be designed to create a refined catalytic microenvironment, enabling simultaneous modulation of the binding strengths of various reactive species. Therefore, Liu *et al.* (*140*) prepared amine-functionalized Ag nanoparticles (NH_2_BPA-Ag) by using 4-aminobutylphosphonic acid (NH_2_BPA), a ligand with both amino and phosphonic acid groups. This catalyst achieved a CO FE of 82% in an H-cell, representing a 2.6-fold improvement compared to pristine Ag nanoparticles. In this work, the amino groups facilitated CO_2_ activation and stabilized intermediates on the catalyst surface, while phosphonic acid groups inhibited the ORR by dissociating the *OOH intermediate. Besides, the physiosorbed phosphonic acid groups with negative charges enhanced the local electric field of EDL by trapping abundant cations through electrostatic interactions, synergistically promoting catalytic performance. However, small amine molecules incorporated into the electrode surface would typically desorb and be removed from the electrode surface in practical electrolysis, leading to an unstable catalytic microenvironment (*141*). For this challenge, polyamines with larger molecule sizes and multiple sp^3^-N centers provide a promising scenario. On this basis, Chen *et al.* (*142*) electroplated polyamine on the Cu electrode surface to increase the selectivity for ethylene production. Experimental evidence indicated that sp^3^-N helped to stabilize intermediates, maintain higher CO content, and keep higher surface pH compared with the entrainment of additives containing little or no amine. Furthermore, polyamine incorporation can alter electrode surface reactivity and keep a stable operation at high current densities.

To address challenges such as low CO_2_ concentration, competitive HER, and poor activity and selectivity in gas-phase CO_2_ electrolysis, sp^2^/sp^3^-N molecules demonstrate unique potential in regulating mass transfer, proton delivery, and changing energy barriers with intermediates. sp^2^/sp^3^-N compounds exhibit distinct yet complementary roles in terms of the electronic environment, CO_2_ adsorption behavior, and catalytic efficiency in gas-phase conversion. sp^2^-N structures can enhance electron conductivity by integrating into the π-conjugated system and modulate the electronic environment of metal active sites, improving catalytic efficiency. However, its direct interaction with CO_2_ is relatively weak. In contrast, sp^3^-N compounds feature more localized electronic structures, which probably do not contribute notably to conductivity but enhance CO_2_ adsorption because of their stronger polarity. This facilitates CO_2_ activation and may potentially influence product selectivity. The fundamental understanding of sp^2^/sp^3^–hybridized nitrogen structures and how they could be better designed and managed in each dimension of regulatory roles in this process are important. Future research would probably focus on investigating how different types of amines, with functional groups like alkyl and hydroxyl groups, as well as their steric hindrance effects, influence the chemical microenvironment and the fundamental reaction mechanism.

## CONCLUSIONS AND PERSPECTIVES

In this review, we explore the roles of sp^2^/sp^3^–hybridized nitrogen centers and propose a forward-looking perspective on advancing CO_2_ capture and reduction through the strategic design and optimization of mediating materials and processes. Thus, we begin by introducing the fundamental concept of sp^2^/sp^3^-N compounds, including their definitions, classifications, and mechanisms of interaction with CO_2_. Different types of sp^2^/sp^3^-N compounds follow distinct pathways in their interactions with CO_2_, leading to varied adsorption kinetics and dynamic processes. Then, the roles of sp^2^/sp^3^-N compounds in electrochemical CO_2_ capture are illustrated. The core capture mechanism involves modulating the affinity for CO_2_ to control its capture and release. Strategies to enhance electrochemical CO_2_ capture efficiency include electronic structure tuning, structural stability and solubility engineering, and operating temperature regulation. Following that, we systemically discuss the direct electrolysis of an integrated amine-based CO_2_ scheme that outperforms less energy-intensive steps, system conciseness, and energy efficiency. For an ideal capture absorbent, we propose that several parameters such as viscosity, p*K*_a_ values, and chemical stability should be considered. At its early development, the probable reactive species containing dissolved free CO_2_ and liquid carbamate are discussed. Early research mostly identified dissolved CO_2_ as the primary reactant, while others considered liquid carbamate as a carbon source. Strategies for improving electrolysis performance are also summarized, including electrode modification, electrolyte additives, and device engineering. Last, the regulatory roles of sp^2^/sp^3^–hybridized nitrogen in traditional gas-phase electrolysis are highlighted. Notably, amines with an affinity for CO_2_ can enhance adsorption and mass transfer, regulate proton delivery, and function as cocatalysts. Regarding the scientific issues and fundamental challenges in mediated roles of sp^2^/sp^3^–hybridized nitrogen for electrochemical CO_2_ capture and conversion, we tentatively propose the following aspects that deserve to be particularly studied in the subsequent stage.

1) Advancements in electrochemical CO_2_ capture have shifted the focus from merely identifying suitable redox-active sp^2^-N and sp^3^-N amine structures to the precise modulation of their properties for enhanced performance. Innovative strategies have tackled key challenges such as the low solubility, oxygen sensitivity, and high reaction potentials associated with sp^2^-N, as well as issues like gas formation, slow metal dissolution, and amine volatility in sp^3^-N systems. Despite these advances, notable barriers remain for scaling electrochemical CO_2_ capture to industrial applications. Practical dilute carbon sources, such as flue gas, often contain impurities like SO*_x_* and NO*_x_*, which can irreversibly degrade finely tuned sp^2^-N structures. Similarly, metal impurities may form stable complexes with sp^3^-N amines, reducing their capture capacity. The long-term thermal stability of sp^2^-N compounds, particularly those with complex functional groups, also remains uncertain under extended operational cycling. In addition, the CO_2_ adsorption performance of reduced sp^2^-N structures in industrial settings requires thorough evaluation to ensure sufficient capture rates and high coulombic efficiency, both critical for seamless integration into the overall process cycle. For instance, while the Cu-EDA couple is regarded as one of the most promising pairs in EMAR, EDA itself, as a branched diamine, exhibits substantial limitations that preclude it from being considered a benchmark sorbent. Moreover, the influence of different Cu crystal facets on reducing the chemical potential or enhancing dissolution rates during the capture process remains insufficiently explored. Consequently, extensive research is needed to develop advanced sorbents, optimized electrolytes, and innovative system designs. These efforts are essential to establish electrochemical CO_2_ capture as a viable alternative to conventional thermal scrubbing and regeneration processes.

2) In the realm of gas-phase CO_2_ electrolysis, the strategic incorporation of suitable sp^2^/sp^3^–hybridized nitrogen structures into overall systems holds great potential for enhancing conversion efficiency and warrants further investigation. For catalysts, the research community should probably focus on expanding the repertoire of sp^2^/sp^3^-N structures for modifying catalysts, particularly those targeting high-value products such as ethylene and other C_2+_ compounds. Furthermore, sp^2^/sp^3^-N structures could enable gas-phase CO_2_ electrolysis directly from flue gases, provided that robust N-containing materials with high CO_2_ selectivity and antioxidation stability are developed. Regarding the regulation of the HER, the roles of amines as additives in gas-phase electrolysis and as sorbents in integrated amine electrolytes differ substantially but remain underexplored. Thus, future research should prioritize uncovering the interactions and reactivity of nitrogen with CO_2_ or its intermediates during the direct electrochemical conversion of dilute CO_2_ streams.

3) In the context of integrated CO_2_ capture and electrolysis, enhancing electrolysis efficiency—particularly in terms of product selectivity, energy efficiency, and carbon efficiency—is crucial. Presently, the FE of such systems is hindered by challenges including poor ion conductivity, fast HER kinetics, and unclear fundamental reaction mechanisms. Moreover, carbon loss remains a critical issue, largely due to the formation of liquid species such as (bi)carbonate and carbamate, which can migrate to the anode in AEM reactors, notably reducing carbon efficiency. To address these challenges, several strategies such as catalyst design and electrode modification, electrolyte optimization, and electrolyzer engineering can be adopted. The development of catalysts with optimized binding energies to achieve a balance between intermediate adsorption and desorption is essential. In addition, enhancing the electrode’s porosity or hydrophobicity could improve mass transfer kinetics and reaction efficiency. Electrolyte optimization by incorporating alkaline cations or suitable surfactants may be positive in the electrochemical process. Furthermore, the electrolyte of amine blend to inhibit HER kinetics by adjusting the ammonium identity and activity on the electrode surface should probably be promising, but there is less attention on this topic. These approaches should be carefully tailored to specific amine-based systems, considering structural factors such as steric hindrance or cyclic configurations. Last, greater emphasis should probably be placed on the direct electrolysis of amine-captured diluted CO_2_ sources such as flue gas or contaminated air. This focus could bridge the gap between laboratory research and practical industrial applications, laying the foundation for scalable and efficient CO_2_ conversion technologies.

4) Advanced characterization techniques and computational modeling and simulations should be developed for the underlying reaction mechanism. For instance, in the reactive CO_2_ capture system, the identity of the active reactant and the dynamic transformation of carbon sources—such as dissolved CO_2_, bicarbonate, and carbamate—remain unclear and, in some cases, controversial because of their constant interconversion. In addition, proton sources, including protonated amines, water, and bicarbonate ions, play critical roles for the contribution to the HER and act as intermediates in the CO_2_RR. However, their electron-proton coupling pathways and reaction mechanisms are not well understood. Future research should prioritize uncovering the intricate reaction pathways of both carbon and proton sources. This can be achieved by combining advanced characterization techniques—such as in situ spectroscopy and synchrotron-based methods—with computational modeling and simulations. Such integrated approaches will provide deeper and more direct insights into these processes, paving the way for improved design and optimization of amine-based electrochemical systems.
